# Metabolic changes during cardiac regeneration in the axolotl

**DOI:** 10.1002/dvdy.70020

**Published:** 2025-03-22

**Authors:** Anita Dittrich, Sofie Amalie Andersson, Morten Busk, Kasper Hansen, Casper Bindzus Foldager, Johan Palmfeldt, Asger Andersen, Michael Pedersen, Mikkel Vendelbo, Kirstine Lykke Nielsen, Henrik Lauridsen

**Affiliations:** ^1^ Comparative Medicine Lab, Department of Clinical Medicine Aarhus University Aarhus Denmark; ^2^ Department of Clinical Medicine, Experimental Clinical Oncology Aarhus University Aarhus Denmark; ^3^ Department of Forensic Medicine Aarhus University Aarhus Denmark; ^4^ Orthopaedic Research Lab, Department of Clinical Medicine Aarhus University Aarhus Denmark; ^5^ Research Unit for Molecular Medicine, Department of Clinical Medicine Aarhus University and Aarhus University Hospital Aarhus Denmark; ^6^ Department of Cardiology Aarhus University Hospital Aarhus Denmark; ^7^ Department of Nuclear Medicine and PET‐Center Aarhus University Hospital Aarhus Denmark; ^8^ Department of Biomedicine Aarhus University Aarhus Denmark

**Keywords:** amphibian, glycolysis, heart regeneration, oxidative phosphorylation

## Abstract

**Background:**

The axolotl is a prominent model organism of heart regeneration due to its ability to anatomically and functionally repair the heart after an injury that mimics human myocardial infarction. In humans, such an injury leads to permanent scarring. Cardiac regeneration has been linked to metabolism and the oxygenation state, but so far, these factors remain to be detailed in the axolotl model. In this descriptive study, we have investigated metabolic changes that occurred during cardiac regeneration in the axolotl.

**Results:**

We describe systemic and local cardiac metabolic changes after injury involving an early upregulation of glucose uptake and nucleotide biosynthesis followed by a later increase in acetate uptake. We detect several promising factors and metabolites for future studies and show that, unlike other popular animal models capable of intrinsic regeneration, the axolotl maintains its cardiac regenerative ability under hyperoxic conditions.

**Conclusions:**

Axolotls undergo dynamic metabolic changes during the process of heart regeneration and display a robust reparative response to cardiac cryo‐injury, which is unaffected by hyperoxia.

## Key Findings

- After cryo‐injury and during regeneration the axolotl increased its global oxygen consumption rate and plasma lactate was decreased.
- In the heart specifically, glucose uptake and nucleotide biosynthesis was especially upregulated early after injury, while acetate uptake was increased later on, especially in the border zone.
- Several key factors were also upregulated in the epicardium during regeneration. Finally axolotl heart regeneration was unaffected by hyperoxia.

## 1 INTRODUCTION

The adult mammalian heart has a very limited capacity for cardiomyocyte (CM) renewal, and a myocardial infarction (MI) therefore leads to permanent scarring and reduced function.^1^ In contrast, fetal and early neonatal mammals have a much higher capacity for cardiac repair.^2^, ^3^, ^4^ Furthermore, popular model organisms like zebrafish, newts, and axolotls, even as adults, retain the ability to regenerate injuries as large as up to 50% of the ventricle after cryo‐injury, mimicking a MI.^5^, ^6^ Intrinsic regeneration is considered to occur mainly via mature cardiac cells undergoing a dedifferentiation process, allowing them to proliferate and repopulate the injured area.^7^, ^8^, ^9^ This has been demonstrated in newts and axolotls throughout their use as a regenerative model since the 1970s with the use of markers of proliferation as well as transmission electron microscopy (Supplementary Table 1).

A link between metabolism and regenerative processes is well established,^10^, ^11^, ^12^, ^13^, ^14^ however, the exact mechanisms and whether these can be generalized in a species‐independent manner remain unclear. The heart is uniquely poised to contribute toward understanding metabolic demands in the event of injury. A constant demand for catabolic activity to maintain sufficient cardiac function cannot be abandoned in favor of anabolic processes associated with proliferation and regeneration.^15^, ^16^, ^17^

In general, animals capable of intrinsic regeneration tend to have a lower metabolic rate compared to adult mammals.^18^, ^19^ Several metabolism‐linked hypotheses have been put forward in recent years, including that the developmental loss of regenerative capacity is associated with the development of endothermy and increasing levels of circulating thyroid hormone.^18^, ^20^ Pathways upregulating cardiomyocyte (CM) glycolysis and the pentose phosphate pathway in favor of oxidative phosphorylation (OXPHOS) and fatty acid oxidation (FAO) have been shown to promote the initial process of regeneration.^13^, ^14^, ^21^, ^22^, ^23^, ^24^, ^25^ These findings were associated with the regulation of several specific signaling pathways, such as mTOR,^26^, ^27^ Nrg1/ErbB2,^14^, ^22^ Krüppel‐like factor,^28^ HIPPO‐Yap,^29^, ^30^, ^31^ and Wnt/β‐Catenin.^32^, ^33^, ^34^ In addition, a correlation between reactive oxygen species (ROS) and CM proliferation has been established in both mammals and zebrafish,^11^, ^12^, ^35^, ^36^ showing that while some level of hypoxia can be beneficial to the regenerative response, hyperoxia has detrimental effects, acting at least in part via the HIF1α signaling pathway.^12^, ^37^, ^38^ This relationship has prompted a fundamental hypothesis, that high levels of OXPHOS, resulting in higher levels of ROS, activate the DNA‐damage response, and block CMs from proliferating.^12^, ^35^, ^39^

In this explorative study, we employed a broad approach to uncover a range of metabolic changes that occurred both systemically and locally within the axolotl heart during the process of regeneration following cryo‐injury. We also demonstrated that, unlike mice and zebrafish, heart regeneration was unaffected by hyperoxia in the axolotl.

## 2 RESULTS

### 2.1 Axolotls fully regenerated after cryo‐injury within 120 days

**FIGURE 1:**
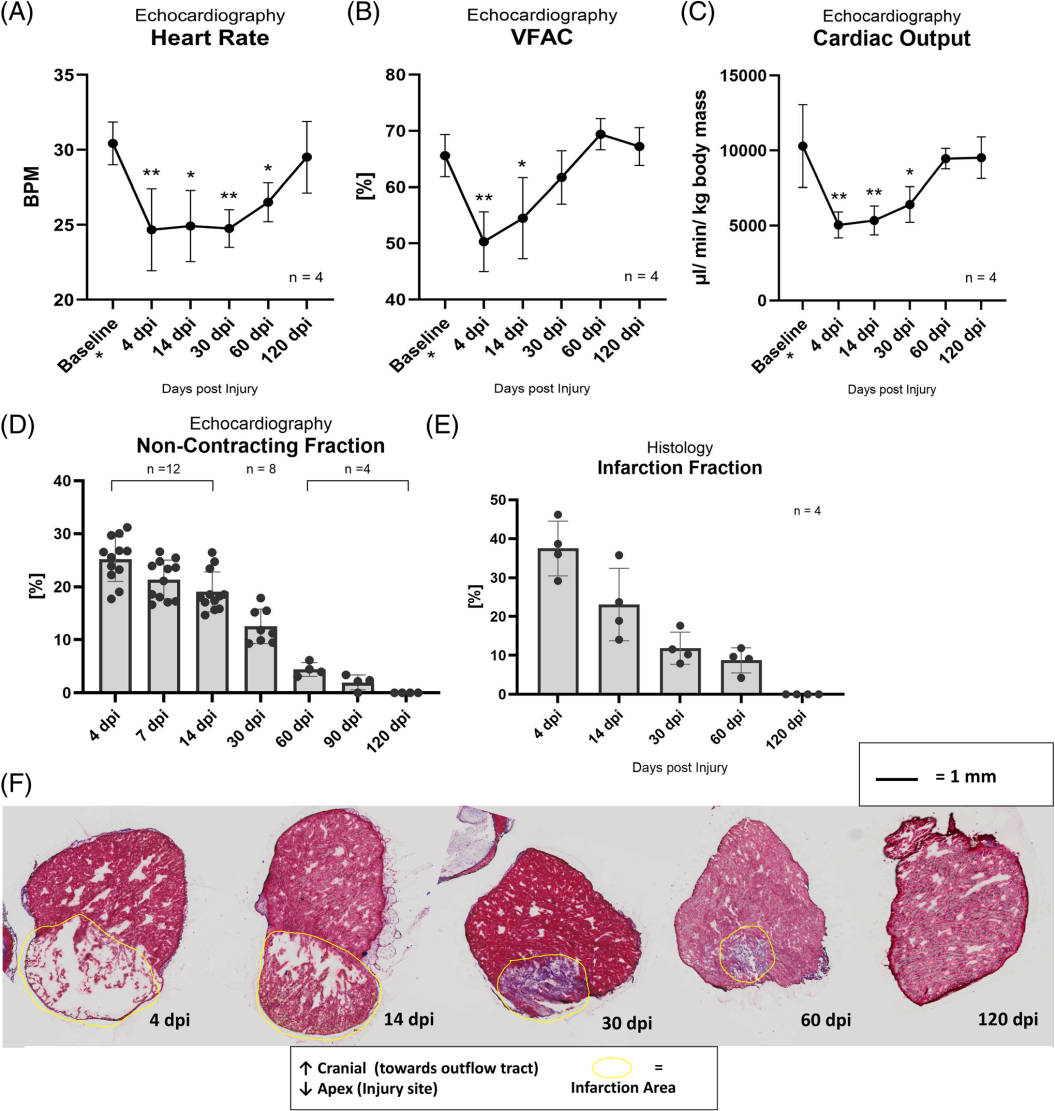
Heart regeneration. (A) Heart rate measured by doppler echocardiography as an average of three cardiac cycles. *n* = 4. (B) Ventricular fractional area change (VFAC) measured by B‐mode echocardiography as an average of three cardiac cycles. *n* = 4. VFAC. (C) Cardiac output measured by B‐mode echocardiography as an average of three cardiac cycles. *n* = 4. (D) Non‐contracting fraction based on echocardiography. The non‐contracting fraction was calculated from area‐tracings as an average across three cardiac cycles. Representative images of echocardiography analysis are shown in Supplementary Figure 1. (E) Infarction fraction based on quantitative stereological analysis of Masson's Trichrome stained cryosections. 10 μm sections obtained at a 150 μm distance were collected throughout the entire ventricle of each heart. (F) Masson's Trichrome stain of cryosections at 4, 14, 30, 60, and 120 dpi. Yellow frame encompasses infarction area. (A–C) was measured in the same four animals that were used for analysis at 120 dpi in (D) and (E). Statistical analysis performed by repeated measures one‐way ANOVA with Dunnet's post hoc test comparing all other time points to baseline in (A–C). *p < 0.05, **p < 0.005. Error bars represent standard deviation.

To evaluate cardiac regeneration, we performed echocardiography and unbiased quantitative histology as previously reported.^40^ After cryo‐injury, cardiac function was affected with a significantly decreased heart rate, ventricular fractional area change (VFAC), and cardiac output compared to baseline (obtained before injury) (Figure 1A–C). One‐way ANOVA with repeated measures showed a significantly decreased heart rate at 4 dpi (days post‐injury) (*p* = 0.0037), 14 dpi (*p* = 0.0053), 30 dpi (*p* = 0.0041), and 60 dpi (*p* = 0.0494), while at 120 dpi, it was no longer different from baseline (Figure 1A). VFAC was similarly different from baseline at 4 dpi (*p* = 0.0015) and 14 dpi (*p* = 0.0179) (Figure 1B). Cardiac output was different from baseline at 4 dpi (*p* = 0.0006), 14 dpi (*p* = 0.0011), and 30 dpi (*p* = 0.0079) (Figure 1C). At 4 dpi, an area without contractile activity was identified with echocardiography, with a mean non‐contractile fraction of 25.22 ± 4.03% (Figure 1D and Supplementary Video 1). The injury decreased in size over time, with no visible non‐contractile fraction remaining at 120 dpi (Figure 1D). This observation was supported by histology with Masson's Trichrome staining (Figure 1E,F), demonstrating that axolotls underwent a similar cardiac tissue regeneration time course in response to cryo‐injury as previously reported,^41^, ^42^, ^43^ which is similar to observations in zebrafish^44^ and neonatal mice.^2^ Functional regeneration can occur before complete anatomical regeneration if the healthy tissue compensates by increasing its contractility (VFAC).

### 2.2 Systemic oxygen consumption rate was upregulated after cryo‐injury, and plasma lactate decreased

**FIGURE 2:**
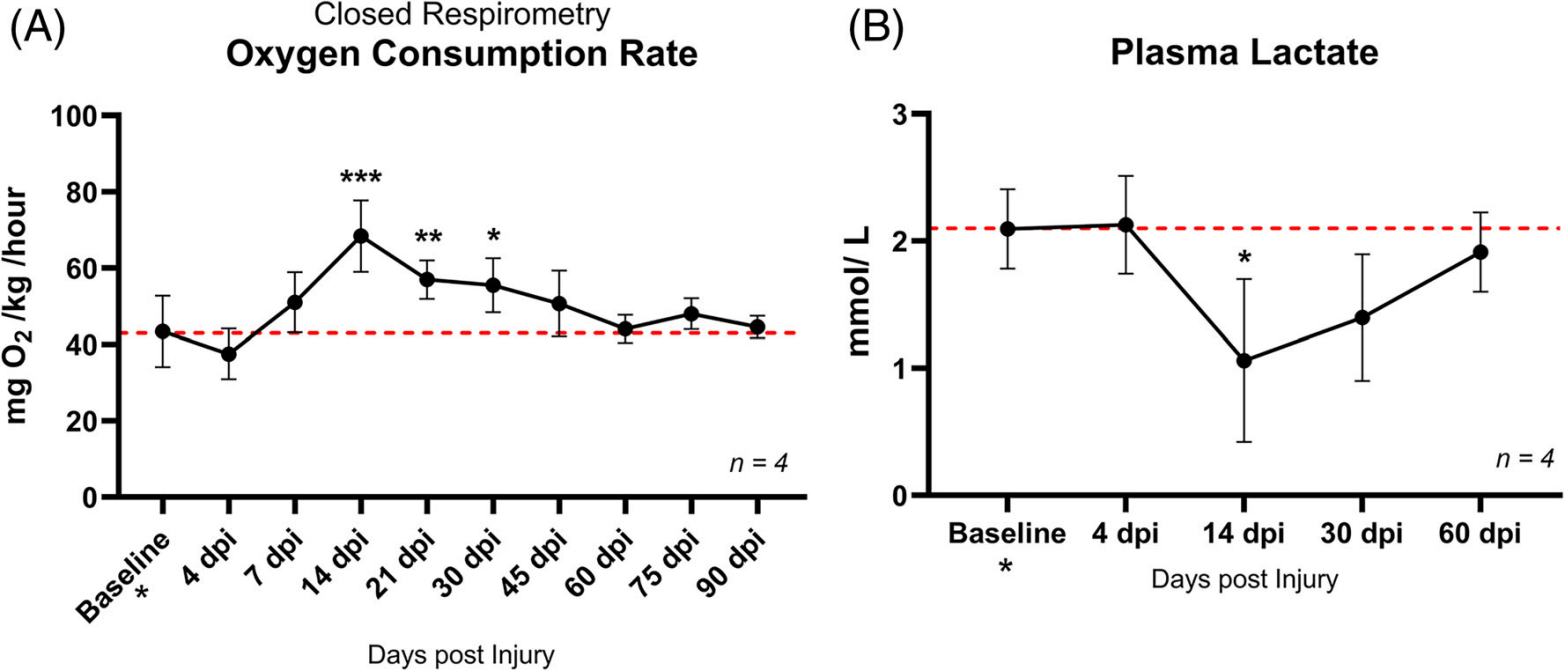
Systemic metabolic changes after cryoinjury. (A) Oxygen consumed during undisturbed closed respirometry. Oxygen was measured using Unisense OXY meter. (B) Plasma lactate assayed with colorimetric microplate assay. All error bars represent standard deviation. Red dotted lines indicate baseline levels. Asterisk represents statistically significant difference from baseline values in one‐way ANOVA with repeated measures followed by Šidák post hoc tests. *p < 0.05, **p < 0.005, ***p < 0.0001.

We first wanted to ascertain whether cryo‐injury had any systemic metabolic effects. Therefore, we measured systemic oxygen consumption using a closed‐respirometry setup. Measurements of oxygen levels before and after 2 h of closed respirometry were obtained from the same individuals (*n* = 4) throughout the regenerative process up to 90 dpi and statistical analysis was carried out by one‐way ANOVA with repeated measures followed by Šidák post hoc tests (Figure 2A). At baseline (prior to injury), axolotls had an oxygen consumption rate of 43.43 ± 8.09 mg O_2_/kg/h. There was a trend toward an increased oxygen consumption rate, starting at 7 dpi followed by a statistically significant peak consumption of 68.42 ± 8.13 mg O_2_/kg/h at 14 dpi (*p* < 0.0001). The O_2_ consumption rate was maintained at a significantly higher level at 21 dpi (*p* = 0.0051) and 30 dpi (*p* = 0.0129) until it returned to a level not significantly different from baseline at 45 dpi and onward (Figure 2A). This data indicates that the axolotl response to cryo‐injury involved a systemic upregulation of oxygen consumption, peaking at 14 dpi. Importantly, this is not a heart‐specific effect in the sense that the heart is necessarily consuming more oxygen, but more likely indicates that other tissues are affected by the loss of cardiac function.

Plasma lactate concentrations (Figure 2B) decreased significantly from a baseline value of 2.09 ± 0.27 mmol/L to 1.06 ± 0.0.55 mmol/L at 14 dpi (one‐way ANOVA with repeated measures followed by Šidák post hoc test, *p* = 0.0381). This suggests a systemic effect of decreased anaerobic metabolism resulting in lower lactate production. Importantly, this, similarly to oxygen consumption, reflects an overall effect across all tissues rather than a heart‐specific metabolic shift.

### 2.3 Hyperoxia led to severely impaired general condition without inhibiting cardiac regeneration

**FIGURE 3:**
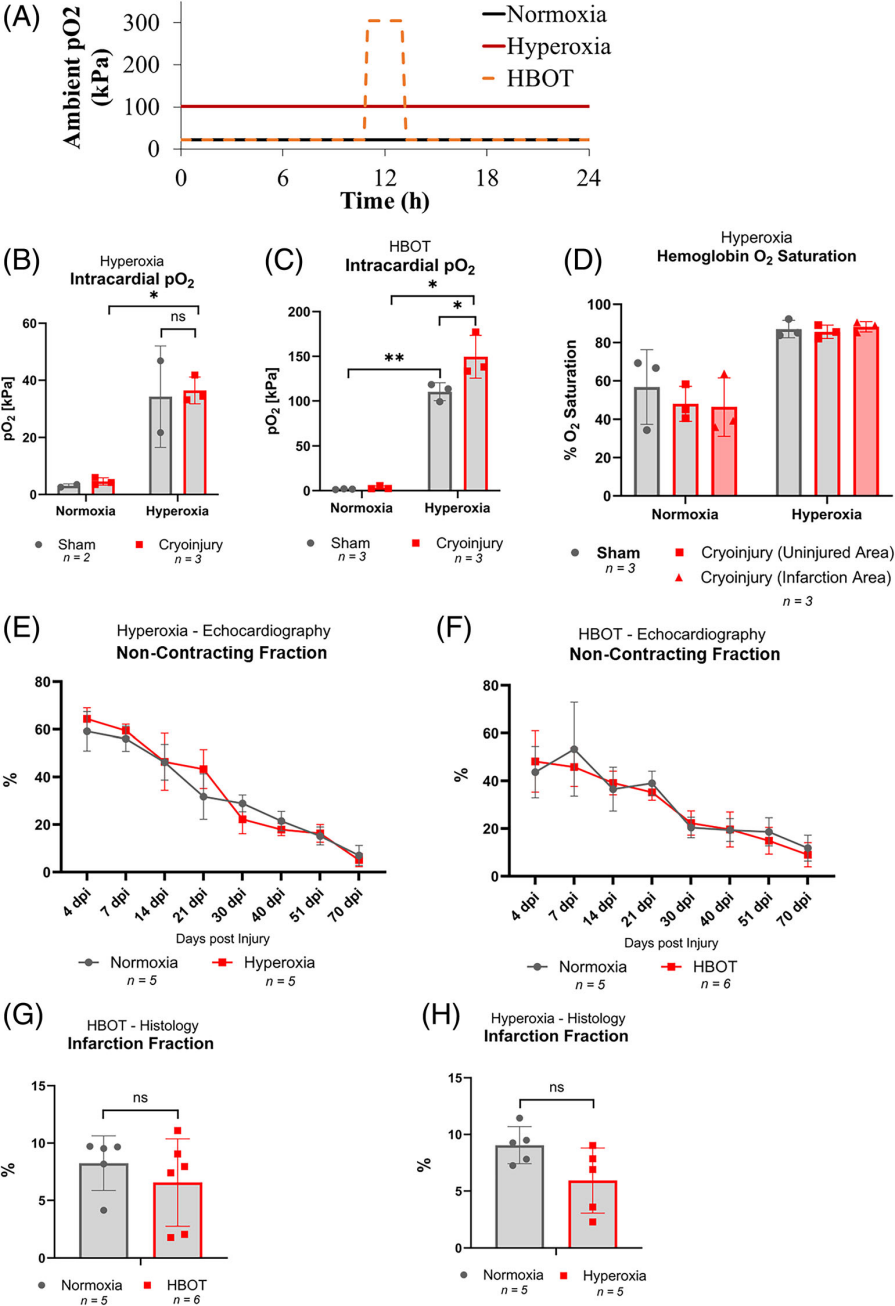
Heart regeneration under hyperoxia. (A) Two types of general hyperoxia treatments were used: constant saturation of housing water with pure O_2_ (constant normobaric hyperoxia) or intermittent hyperbaric O_2_ treatment (HBOT) where animals were exposed to a daily regimen of 2 h 3 atm with complete O_2_ saturation of housing water. (B–C) Intracardiac plasma pO_2_ during normoxia, hyperoxia, and HBOT exposure in sham and cryo‐injured animals at 4 dpi. (D) Intracardiac hemoglobin O_2_‐saturation during normoxia and hyperoxia in sham and cryoinjured animals at 4 dpi. Values for both the uninjured and injured zone of the heart are provided for cryo‐injured animals. (E,F) Non‐contracting fraction measured by echocardiography in the course of 70 days post cryo‐injury in two rounds of experiments including axolotls exposed to normoxia and hyperoxia or HBOT, respectively. (G,H) Infarction fraction measured by quantitative histology at the termination of hyperoxia and HBOT experiments at 73 dpi where there was no significant difference in the remaining fraction of infracted myocardium. Error bars represent standard deviation. For figure elements (B–F), asterisk indicates significant difference in unpaired *t*‐tests, while in (G) and (H), statistics were performed with one‐way ANOVA with repeated measures and multiple comparisons between groups and time points with Šidák post hoc tests (ns = non‐significant difference between groups at all time points). *p < 0.05 and **p < 0.005.

With hyperoxia and ROS being suggested as the main factors capable of limiting heart regeneration,^12^, ^35^, ^39^ we wanted to test whether hyperoxia would have any inhibitory effect on axolotl heart regeneration. In general, hyperoxia treatment led to severe impairment of axolotl well‐being in our study. O_2_‐treated animals suffered from general lethargy, stiffness, and lack of muscle function. Both constant normobaric hyperoxia and intermittent hyperbaric oxygen treatment (HBOT) (Figure 3A) led to an increase in intracardiac plasma pO_2_ in both sham and cryo‐injured animals at 4 dpi (constant hyperoxia: unpaired *t*‐tests of normoxia vs. hyperoxia not possible in sham group with only 2 replicates, *p* = 0.0089 in cryo‐injured group; HBOT: unpaired *t*‐tests of normoxia vs. HBOT, *p* = 0.0026 in sham group and *p* = 0.0075 in cryo‐injury group) (Figure 3B,C). At normoxia, hemoglobin was ~50% saturated with O_2_ at 4 dpi in healthy sham and cryo‐injured animals, and this level was elevated to close to 100% saturation during exposure to normobaric hyperoxia (Figure 3D). Thus, during hyperoxia treatment, cardiac tissue was exposed to a markedly increased level of O_2_, resulting from both completely O_2_‐saturated erythrocytes and blood plasma with higher O_2_ concentration. However, despite a general decrease in animal well‐being during elevated exposure to O_2_, there was no significant effect on regenerative capacity (Figure 3E–H). Non‐contracting fraction in both types of O_2_ treatment, as estimated by echocardiography, decreased over time in a similar fashion as in normoxia and hyperoxia/HBOT‐treated animals (one‐way ANOVA with repeated measures with multiple comparisons between groups and time points with Šidák post hoc tests found no significant difference between normoxia vs. hyperoxia or normoxia vs. HBOT at any time points) (Figure 3E,F). Likewise, quantitative histology revealed no significant difference in infarction fraction using unpaired *t*‐tests (Figure 3G,H). Further research is required to understand these rather unexpected results showing that axolotls possess an ability to perform cardiac regeneration in the presence of hyperoxia.

### 2.4 Glucose uptake was increased throughout the ventricle during the early time point and later downregulated after injury

**FIGURE 4:**
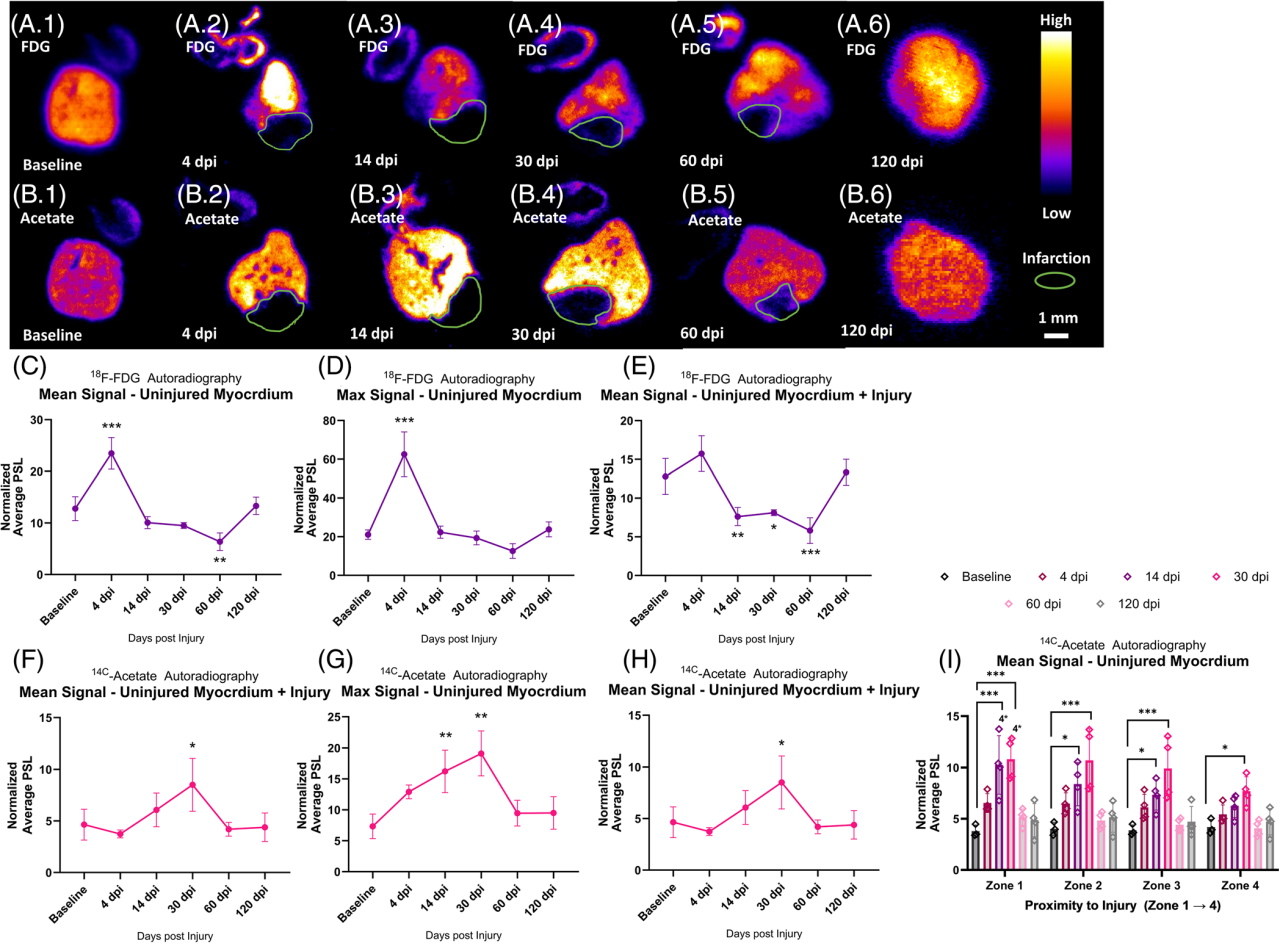
Autoradiography. (A) Representative images of ^18^F‐FDG autoradiography imaged cryosections at baseline, 4, 14, 30, 60, and 120 dpi. (B) Representative images of ^14^C‐acetate autoradiography imaged cryosections at baseline, 4, 14, 30, 60, and 120 dpi. (C) Average ^18^F‐FDG signal in uninjured myocardium. (D) Maximum ^18^F‐FDG signal in uninjured myocardium. (E) Average ^18^F‐FDG signal across entire ventricle including infarction area. (F) Average ^14^C‐acetate signal in uninjured myocardium. (G) Maximum ^14^C‐acetate signal in uninjured myocardium. (H) Average ^14^C‐acetate signal across the entire ventricle including infarction area. (I) Average signal of ^14^C‐acetate in different zones (zone 1 closest to infarction, zone 4 furthest away. All error bars represent standard deviation. Asterisks indicate statistical significance in one way ANOVA with Šidák post hoc test. *p < 0.05, **p < 0.005, and ***p < 0.0001. 4* indicates significant difference from zone 4. *n* = 3 in baseline group and *n* = 4 in all other groups. Corresponding brightfield images of the sections are shown in Supplementary Figure 3.

To also specifically assay cardiac metabolism rather than systemic metabolism in response to injury, we employed autoradiography using radiotracers. ^18^F‐FDG is a glucose analogue, and its uptake is therefore a proxy measurement of glucose uptake. We found a significant increase in ^18^F‐FDG uptake in the uninjured myocardium of the ventricle at 4 dpi (one‐way ANOVA with Šidák post hoc test, *p* < 0.0001) (Figure 4A,C), with the average normalized photo‐stimulated luminescence (PSL) value almost doubling from a mean of 12.08 ± 1.89 at baseline to 23.49 ± 2.64 at 4 dpi. The signal returned to baseline levels by 14 dpi and remained low, with values at 60 dpi significantly lower compared with baseline (one‐way ANOVA with Šidák post hoc test, *p* = 0.0027) but no longer statistically different from baseline at 120 dpi. The maximum normalized PSL within the uninjured myocardium likewise increased from 21.12 ± 1.99 at baseline to 62.58 ± 9.95 at 4 dpi (one‐way ANOVA with Šidák post hoc test, *p* < 0.0001) (Figure 4D). When measuring the average signal across the entire ventricle (i.e., including the infarction area) there was no longer a significant difference between baseline and 4 dpi (Figure 4E), but rather a decrease at 14, 30, and 60 dpi (one‐way ANOVA with Šidák post hoc test, *p* = 0.0049 (14 dpi), *p* = 0.0106 (30 dpi), and *p* = 0.0003 (60 dpi)). This indicated that glucose uptake mechanisms were locally upregulated in the remaining uninjured myocardium in response to injury at the early time point of 4 dpi but later decreased to below baseline values before returning to normal; thus, glucose uptake was differentially regulated across the regenerative timeline. The signal did not display any apparent border‐zone effect.

### 2.5 Acetate uptake was upregulated most prominently in the border zone at 14–30 days after injury

When ^14^C‐acetate is taken up by cells, it is metabolized to acetyl‐CoA, which is either shuttled into the tricarboxylic acid (TCA) cycle for energy production or utilized in lipid biosynthesis to produce new cell membranes needed for proliferating and/or growing cells. While glucose uptake peaked at 4 dpi, increased uptake of acetate appeared later, increasing gradually and peaking at 30 dpi before returning to baseline values by 60 dpi (Figure 4B,F–H).

Within the uninjured myocardium, the average normalized signal value increased from baseline 4.65 ± 1.21 to 10.24 ± 2.46 at 30 dpi (one‐way ANOVA with Šidák post hoc test, *p* = 0.0023) (Figure 4F). The maximum normalized signal value also peaked at 30 dpi with a normalized maximum signal value of 19.10 ± 3.11 compared to 7.34 ± 1.61 at baseline (one‐way ANOVA with Šidák post hoc test, *p* = 0.0002) (Figure 4G). When averaging across the entire ventricle, there was also a significant peak at 30 dpi (one‐way ANOVA with Šidák post hoc test, *p* = 0.0236) (Figure 4H). This means that unlike ^18^F‐FDG uptake at 4 dpi, ^14^C‐acetate uptake at 30 dpi rose to levels above that of the intact ventricle as a whole at baseline during the response to injury. Furthermore, the acetate signal appeared to be more prominent in the border zone. To quantify the acetate signal in relation to the injury site, we divided the ventricle into zones, with zone 1 being closest to the injury and zone 4 furthest away (Supplementary Figure 4), and found using a linear mixed model with Tukey post hoc tests that in zones 1, 2, and 3, there was a significantly higher signal compared to baseline values at 14 and 30 dpi (Figure 4I). Only at 30 dpi was there also a difference from baseline in zone 4. When comparing the signal across zones at 14 and 30 dpi, there was also a significant difference between zones 1 and 4.

### 2.6 Metabolomics analysis identified more than 200 metabolites

**FIGURE 5:**
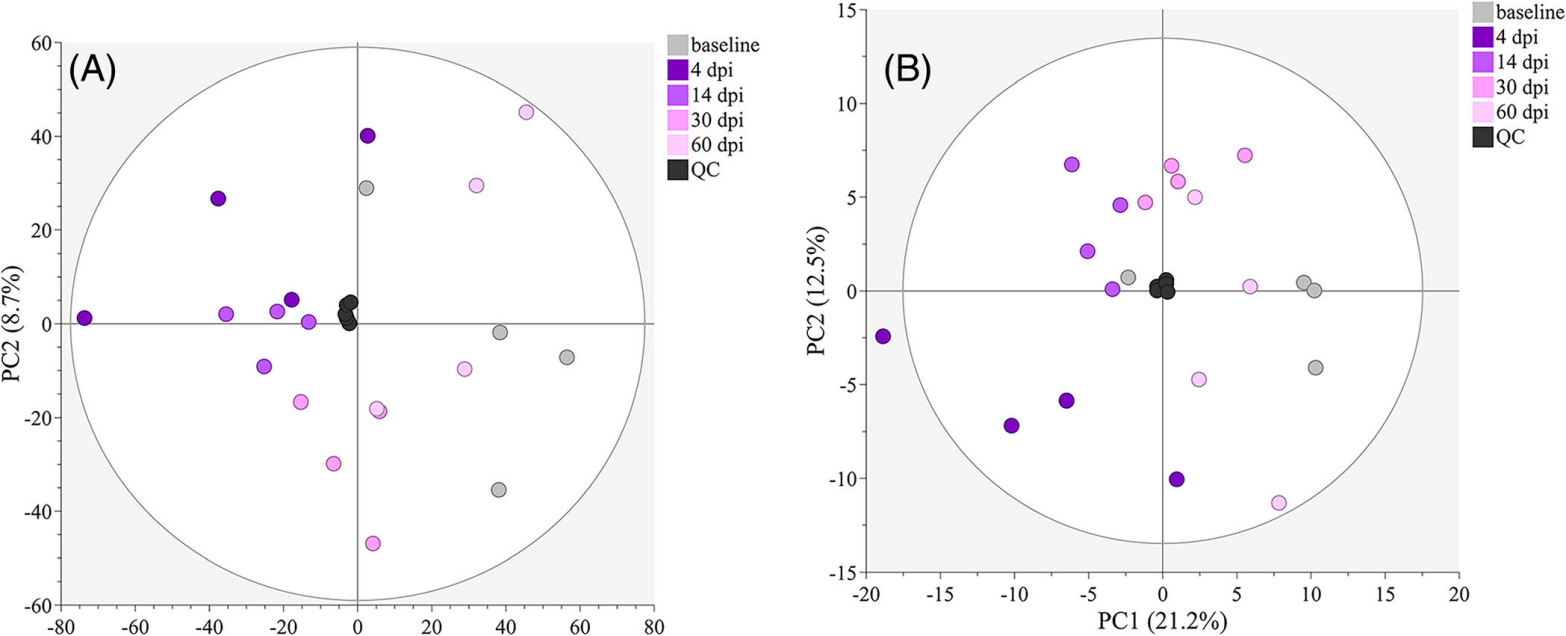
Metabolomics PCA scores plot. (A) All detected features in positive and negative ionization mode following pre‐processing and normalization. (B) All annotated and identified metabolites following normalization. QC = quality controls. Quality controls are the same pooled sample injected for each fifth sample. QCs are in the center of the PCA scores plot, and with low variation, indicating that the analysis was not affected by instrumental errors. The circle indicates Hotelling's *T* ^2^. *n* = 4 in all groups.

To further evaluate cardiac‐specific effects, we performed shotgun metabolomics on whole ventricles using liquid chromatography‐mass spectrometry. Metabolites are diverse and susceptible to either negative or positive ionization in mass spectrometry. We wanted to capture as many metabolites as possible and therefore applied both ionization modes. Following preprocessing, 4354 features were found in positive ionization mode and 1233 features were found in negative ionization mode. Principal component analysis (PCA) indicated that the main differences from baseline were at 4 dpi (Figure 5), and largely included metabolites involved in nucleotide biosynthesis pathways and the glutathione redox system. We therefore focused our statistical analysis on these time points and nucleotide biosynthesis‐ and glutathione‐pathways. In total, 204 metabolites were identified and annotated and used for further analysis (of which 52 had a *p*‐value of <0.05 when comparing baseline and 4 dpi in unpaired *t*‐tests). A complete table of results from metabolomics analysis is presented in Supplementary Table 2. We did not detect many metabolites associated with glycolysis or oxidative phosphorylation, which may be due to the stability of these metabolites and/or the specific solvent used. The protocol was optimized to capture as wide a variety of metabolites as possible without bias. It is a limitation that the metabolomics data are broad and untargeted. However, the criteria for metabolomics data treatment were stringent as described in Supplementary Methods, and they can be highly valuable as descriptive and hypothesis‐generating data.

### 2.7 Anabolic pathways for nucleotide biosynthesis were upregulated at 4 dpi

**FIGURE 6:**
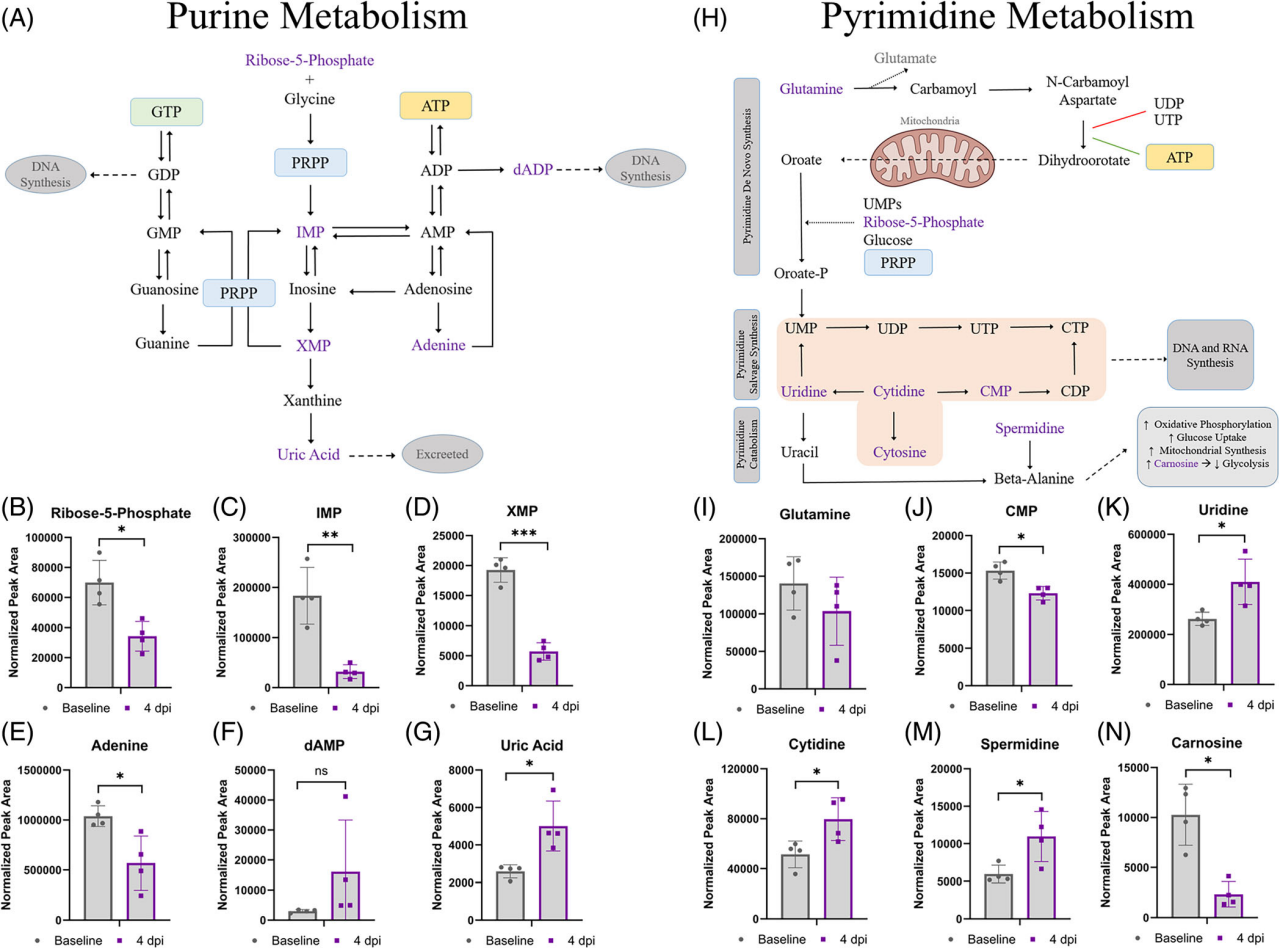
Nucleotide metabolism. (A) Pathway map of purine metabolism. Metabolites in purple were detected by metabolomics in cardiac tissue samples. (B–G) Metabolic profiles of d‐ribose‐5‐phosphate, inosine monophosphate (IMP), xanthosine monophosphate (XMP), adenine, deoxyadenosine monophosphate (dAMP), and uric acid, respectively. (H) Pathway map of pyrimidine and beta‐alanine metabolism. Metabolites in purple were detected by metabolomics in cardiac tissue. (I–P) Metabolic profiles of glutamine, cytidine monophosphate (CMP), uridine, cytidine, spermidine, and carnosine, respectively. All error bars represent standard deviations. Asterisk indicates statistical significance in an unpaired *t*‐test comparing baseline and 4 dpi samples. *p < 0.05, **p < 0.005, and ***p < 0.0001. *n* = 4 in all cases.

Metabolites from the purine metabolism pathway were measured as nominally changed at 4 dpi compared with baseline (*p* < 0.05) (Figure 6A–G). d‐Ribose 5‐phosphate (R5P), IMP, XMP, and adenine were decreased at 4 dpi. Conversely, uric acid was increased at 4 dpi. Overall, these findings indicated increased consumption of precursors of nucleotides for DNA synthesis. In the pyrimidine pathway, we found a non‐significant tendency of decreased glutamine, which is the source of nitrogen required to initiate both purine and pyrimidine synthesis. We also found a statistically significant decrease in CMP at 4 dpi, while uridine and cytidine were increased (Figure 6H–N). Carnosine was decreased. Spermidine, which was elevated at 4 dpi, is further described in Table 1 along with other metabolites that have shown promise as pro‐regenerative substances in other contexts and might represent valid candidates for future study as possibly pro‐regenerative metabolites in the axolotl. Overall, these results suggest an increased demand for pyrimidine synthesis at 4 dpi. The decrease in R5P along with an increase in glycerate (Supplementary Table 2) furthermore indicates utilization of these metabolites for the pentose phosphate pathway.

**TABLE 1:** Metabolites with known links to regeneration in mammalian models.

Metabolite (upregulated at 4 dpi vs. baseline)	Role in regeneration
5′‐methyl‐thioadenosine (MTA)	Involved in signaling pathways regulating gene expression, proliferation, differentiation, and cell death, and inhibits synthesis of spermidine.^45^
Betaine (detected, but not significant)	Antioxidant shown to improve wound healing^46^ and promotes liver regeneration and synthesis of spemidine.^47^ N‐N‐N‐trimethyl lysine which is structurally highly similar to betaine was also upregulated at 4 dpi (Supplementary Table 2).
Cytidine	A synthetic form of cytidine can lessen adverse cardiac remodeling after an MI.^48^
Spermidine	Enhances autophagy, reduces ROS damage, reduces CM apoptosis, inhibits the mTOR pathway and improves outcome from MI in rats.^49^ Other studies showed spermidine to have cardioprotective effects and enhance the lifespan of mice.^50^, ^51^
UDP‐glucose	Converts galactose to glucose. Presence of galactose is associated with aging of the heart.^52^
Uridine	Has pro‐regenerative capacities in several species and tissues.^53^

*Note*: Complete results in Supplementary Table 2.

### 2.8 Alterations in glutathione metabolites indicated a response to oxidative stress after cryoinjury

**FIGURE 7:**
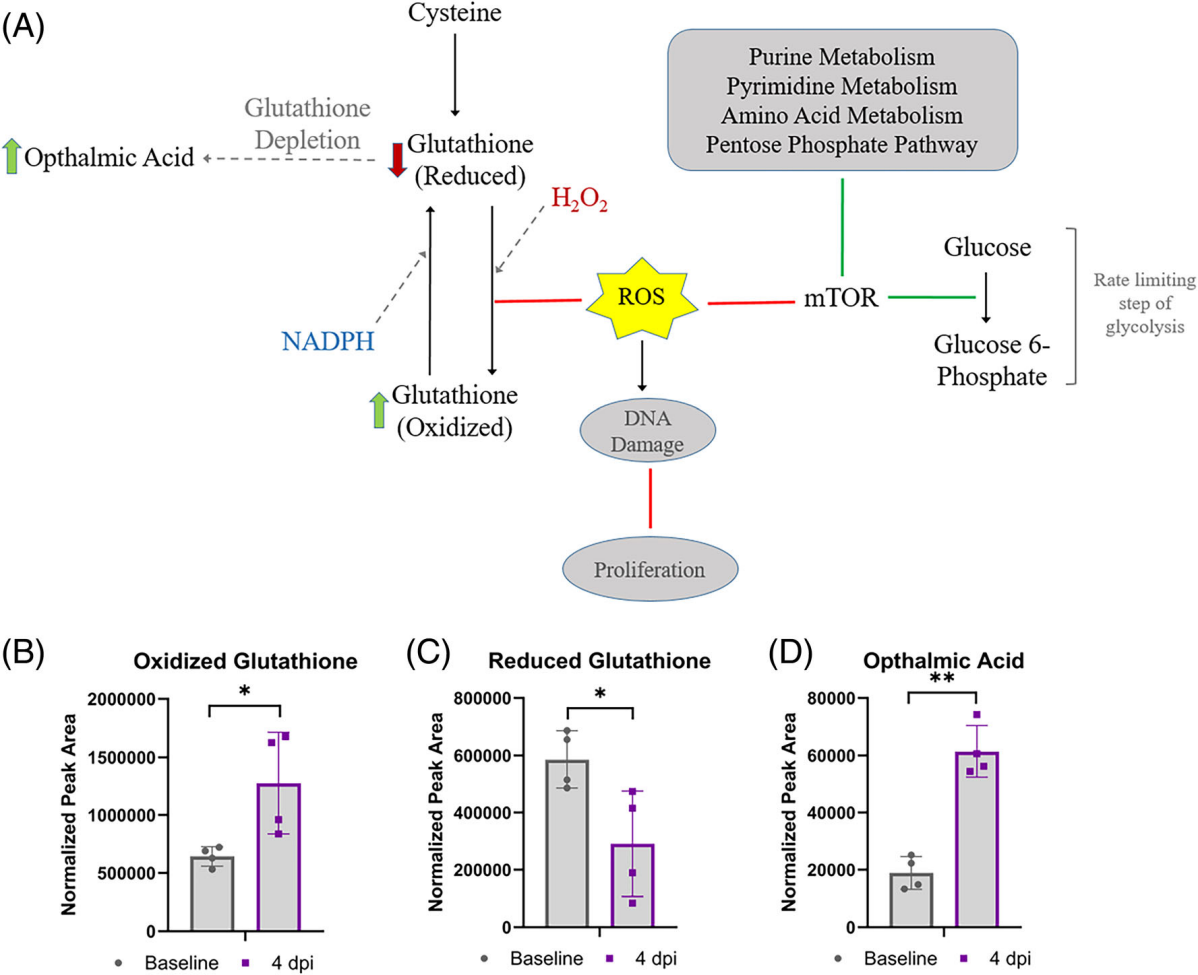
Glutathione metabolism. (A) Pathway map of glutathione metabolism and connection to other pathways. (B–D) Metabolomics profile of reduced glutathione, oxidized glutathione, and ophthalmic acid at baseline at 4 dpi. Asterisk indicates *p*‐value <0.05 in unpaired *t*‐test comparing baseline at 4 dpi samples. All error bars represent standard deviations. Asterisk indicates statistical significance in an unpaired *t*‐test comparing baseline and 4 dpi samples. *p < 0.05 and **p < 0.005. *n* = 4 in all cases.

With a significant decrease in the antioxidant glutathione (reduced form) at 4 dpi compared to baseline, along with an increase in the oxidized form of glutathione (Figure 7B,C), we found that cryo‐injury was associated with changes in the glutathione redox balance at 4 dpi, with putative links to oxidative metabolism and the presence of reactive oxygen species (ROS) (Figure 7A). This was also supported by a rise in ophthalmic acid (Figure 7D). Ophthalmic acid is formed when the precursor to produce glutathione, cysteine, becomes limited.^54^, ^55^ This indicates that although axolotl heart regeneration was remarkably resistant to the expected negative effects of hyperoxia, axolotls are not counteracting any and all shifts in redox balance.

### 2.9 mTOR activity was upregulated in the heart after cryo‐injury

**FIGURE 8:**
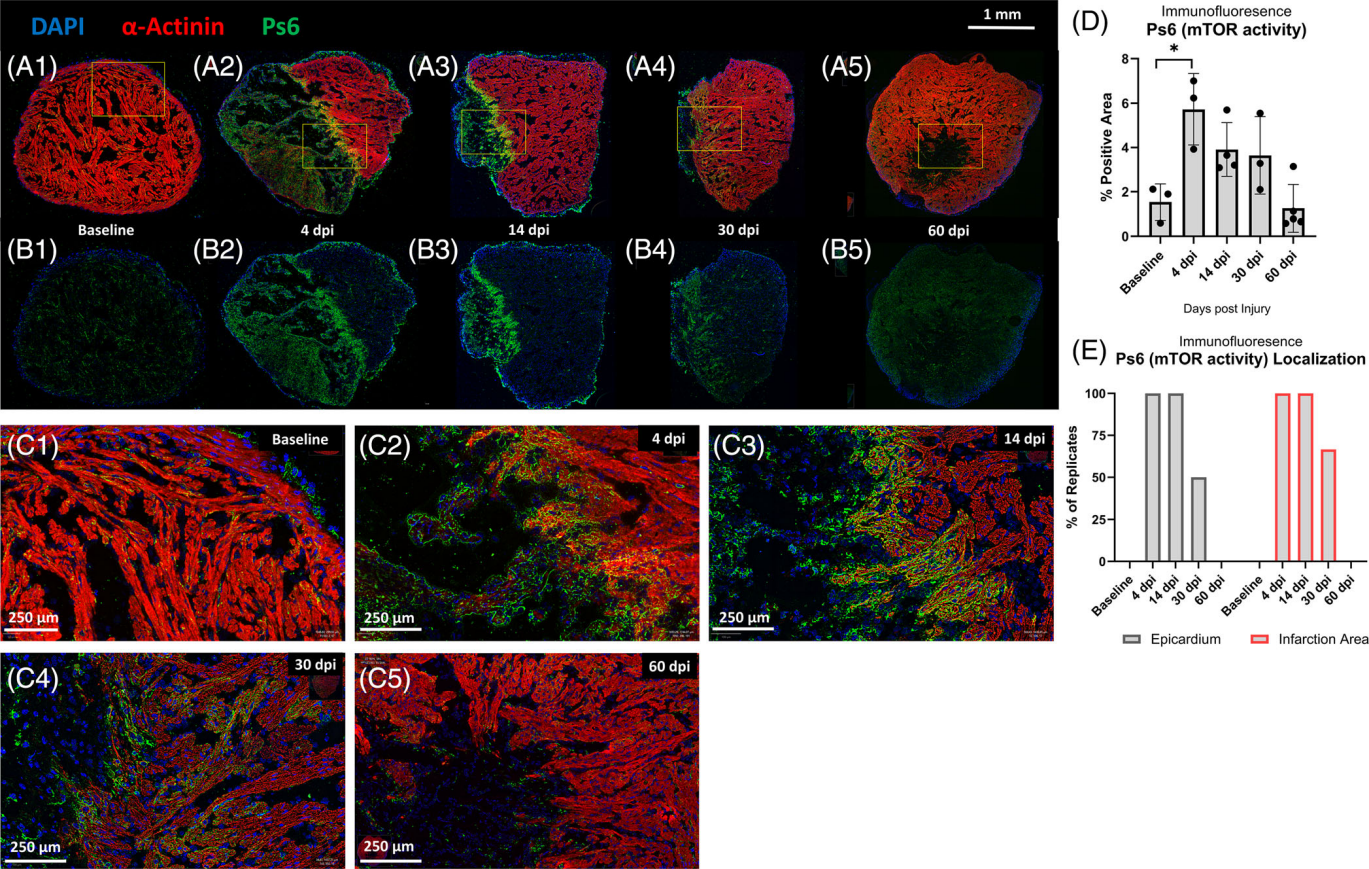
mTOR activity in the heart after cryo‐injury. (A–E) Immunofluorescence labeling of Ps6 (Serine‐6‐phosphorylated ribosomal protein, a known marker of mTOR activity). (A) 7 μm cryosections stained for Ps6 (FITC, Green), α‐actinin (Cy5, Red), and DAPI nuclear stain (Blue). Areas that are not α‐actinin‐positive represent the remaining injury. (B) Same images as above without red channel to show Ps6 activity in the entire ventricle. (C) Higher magnification images corresponding to yellow brackets in (A). (D) Semi‐quantitative analysis of Ps6 positivity. The fractional area positive for Ps6 above a standardized threshold and laser intensity measured across three sections from each animal in QuPath. All error bars represent standard deviation. Asterisk indicates *p*‐value <0.05 in one‐way ANOVA with Šidák post hoc test comparing each group to baseline. (E) Percentage of animals in each group where in all three sections Ps6 activity was found to be localized in and around the infarction area and/ or the epicardium. Brightfield images of neighboring sections in Supplementary Figure 5.

mTOR signaling is an evolutionarily conserved pathway that couples environmental conditions, including stress and nutrient levels, with cellular growth and proliferation.^56^ It is also known to be inhibited by both high levels of ROS and spermidine in other model organisms.^57^ We performed double‐immunofluorescence against Ps6 (serine‐6‐phosphorylated ribosomal protein), a known marker of mTOR activity,^58^, ^59^ and α‐actinin, a marker of mature healthy cardiomyocytes. The antibody for Ps6 has previously been used to show that activated cells in the axolotl limb after amputation injury display activated mTOR signaling during limb regeneration.^60^ Considering this, we wanted to assay whether heart regeneration was similarly associated with an increase in mTOR activity with possible downstream effects on metabolism. We found that mTOR activity was upregulated after injury (Figure 8A–E) and concentrated at the site of injury, the border zone area as well as the epicardium, especially at 4 and 14 dpi. While at baseline and the later time points after cryo‐injury, the signal was more evenly distributed throughout the ventricle (Figure 8E). Averaging three sections from each heart and measuring the fraction of Ps6 positive area relative to the entire ventricle, baseline ventricles were found to have a positive area fraction of 1.53 ± 0.67%. In 4 dpi hearts, this was significantly increased to 5.72 ± 1.31% (one‐way ANOVA with Šidák post hoc tests, *p* = 0.0064) before gradually returning to baseline levels by 60 dpi (Figure 8D). This may indicate that some cells within the epicardium and in and around the injury site become activated during the early stages of regeneration in a similar way to what has been observed in the axolotl limb regeneration model.^60^ Importantly, we cannot rule out that this signal is at least in part originating from immune cells.

### 2.10 Enzyme activities were different in the injured and uninjured areas

**FIGURE 9:**
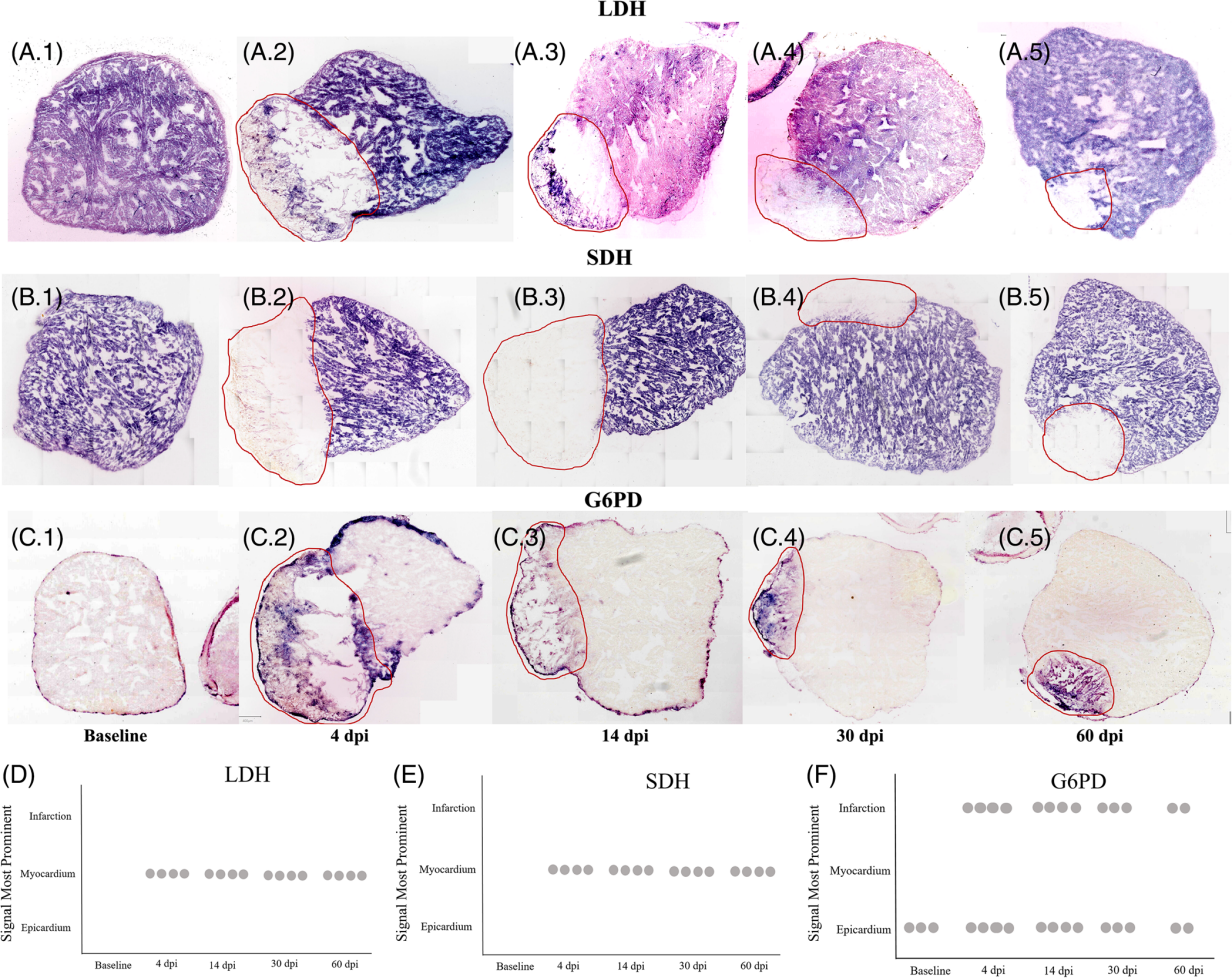
Enzyme histochemistry stains. Deposition of blue product from nitroblue tetrazolium chloride reaction indicates enzymatic activity in the presence of substrate (here either LDH, SDH, or G6PD). The red lines indicate the injured area recognized based on morphology of the hematoxylin counter stain (counter stain appears faint in the images due to the much higher saturation of enzyme specific stain). (A) LDH activity enzyme stain, (B) SDH activity enzyme stain, and (C) G6PD activity enzyme stain. (D–F) Each dot represents a replicate (heart) in which three sections all show staining pattern where signal is most prominently localized to the designated area (infarction, myocardium, or epicardium) for LDH, SDH, and G6PD, respectively.

Last, we performed dehydrogenase enzyme histochemistry for three key enzymes—lactate dehydrogenase (LDH), succinate dehydrogenase (SDH), and glucose‐6‐phosphate dehydrogenase (G6PD). These represent key metabolic pathways that we were interested in. Lactate is an important fuel source for the mammalian myocardium, and reduced activity of LDH is a sign of cardiac pathology.^61^ Also, LDH converts lactate to pyruvate and vice versa and is critical to anerobic metabolism. Further, we wanted to know if anaerobic metabolism was activated in response to injury in the axolotl. We found LDH activity to be evenly distributed throughout the myocardium at baseline (Figure 9A,D). LDH activity remained present after injury throughout the uninjured part of the myocardium, and while activity was diminished in the injured area, some activity remained, especially in the outer edge at 4 and 14 dpi, indicating that anaerobic metabolism is utilized by the axolotl heart both at baseline and after injury. Importantly, the signal localized to the injury site could also stem from immune cells in the area.

SDH is a mitochondrial membrane‐bound enzyme that shuttles TCA metabolites into the respiratory chain and is thus an indicator of OXPHOS. Within the uninjured myocardium, SDH activity levels resembled LDH activity levels, being prominent throughout the uninjured myocardium and remaining so throughout regeneration. Within the injury site, on the other hand, SDH activity was almost completely abolished (Figure 9B,E).

G6PD converts glucose‐6‐phosphate to 6‐phosphogluconolactone, which shuttles available glucose into the pentose phosphate pathway and plays an important role in maintaining a pool of reduced glutathione.^62^ The pentose phosphate pathway has also been shown to be critical to tissue regeneration.^63^, ^64^ Intriguingly, G6PD activity was generally low within the uninjured myocardium but prominent in the epicardium at baseline. After injury, G6PD activity was increased within the injured area, suggesting a localized shift toward pentose phosphate pathway activity (Figure 9C,F). Similarly to LDH, the G6PD signal within the injury site could very well stem from immune cells.

## 3 DISCUSSION

In this explorative study, overall, we found that several metabolic changes occurred during cardiac regeneration, both systemically and locally within the heart after cryo‐injury. On a systemic level, O_2_ consumption was upregulated, peaking at 14 dpi before returning to baseline levels by 45 dpi (Figure 2A), suggesting an overall increase in oxidative metabolism at this time. Also, at 14 dpi, plasma lactate was decreased, which simultaneously suggests a systematic decrease in anaerobic metabolism (Figure 2B). These responses may be an integral part of a more general systemic activation to injury, like the systemic cell activation that has been documented in axolotls after limb amputation.^60^ Importantly, this does not pertain to cardiac metabolism in isolation, and a metabolic shift in the heart is not necessarily mirrored by other tissues. With respect to O_2_ consumption, it is also important to note that axolotls can obtain O_2_ via the skin, gills, and lungs, meaning reduced cardiac function could be offset by increased dermal absorption of O_2_.

Surprisingly, systemic hyperoxia had no negative impact on cardiac regeneration in the axolotl (Figure 3). Even though lethargic behavior and muscle stiffness were observed, and we could detect elevated levels of intracardial pO_2_ (Figure 3B,C) along with hemoglobin O_2_ saturation (Figure 3D) in response to both chronic and intermittent hyperoxia, neither treatment had any impact on the regenerative process after cryo‐injury (Figure 3E–H). This finding warrants further study as axolotls may employ some cardio‐protective mechanism when exposed to high levels of O_2_, and thus elevated ROS. The harmful effects of hyperoxia and ROS in the context of heart regeneration have otherwise been emphasized in recent studies on heart regeneration across other species.^11^, ^12^, ^35^, ^36^, ^39^

In the heart tissue, we found an indication that a response to ROS was ongoing at 4 dpi under normoxic conditions, as the ratio of reduced vs. oxidized glutathione was decreased along with an upregulation of ophthalmic acid (Figure 7), which tends to increase when antioxidant capacity for glutathione reduction becomes limited.^54^ Future studies should explore how the glutathione redox state as well as other relevant markers of downstream effects of increased ROS are affected by injury as well as hyperoxia in the axolotl. It would also be important to address whether shifts in redox balance lead to increased DNA damage in the axolotl, along with a more general investigation into protective mechanisms against DNA damage in this model. The glutathione system plays a role in regulating the overall levels of ROS, and ROS can in turn, among its many effects, inhibit the mTOR pathway, which is a potential key pathway regulating metabolism and proliferation.^26^, ^57^, ^65^, ^66^, ^67^, ^68^, ^69^, ^70^ Spermidine was upregulated at 4 dpi and could be a potential candidate for factors with a protective mechanism against ROS^49^, ^50^ (Table 1 and Figure 6). The other metabolites with known pro‐regenerative effects, like spermidine, that were upregulated in the axolotl heart at 4 dpi are also uncharacterized in terms of their specific roles in intrinsic cardiac regeneration in the axolotl. These included 5′‐methyl‐thioadenosine, N‐N‐N‐trimethyl lysine, cytidine, UDP‐glucose, and uridine (Table 1).

Using autoradiography, we observed that the uptake of the glucose analogue ^18^F‐FDG was elevated throughout the remaining uninjured myocardium at the early 4 dpi timepoint, seemingly without any border‐zone effect (Figure 4A,C–E). An integral and beneficial role of glycolysis‐driven metabolism in the context of heart regeneration, especially early after injury, has been widely documented in a range of species, including mice, pigs, and zebrafish,^14^, ^21^, ^22^, ^24^, ^25^ and seems also to be the case in the axolotl. Due to our limited number of time points, it is not clear how long this increased glucose uptake lasts. Intracellular glucose enters the glycolytic pathway, where it is metabolized to pyruvate, which enters the mitochondria for oxidative catabolism in the TCA cycle and respiratory chain or is metabolized to lactate by LDH (activity of which was upregulated in and around the injury at 4 and 14 dpi [Figure 9A,D]). LDH can facilitate anabolic metabolism, which can produce key intermediates for the TCA cycle as well as the pentose phosphate pathway. Notably, LDH activity within the injury itself may stem from immune cells.

Glycolytic metabolites can alternatively be shuttled into the pentose phosphate pathway, which has also been shown to be beneficial to cell proliferation and regeneration,^23^, ^64^, ^71^ especially due to its importance in generating precursors for nucleotide biosynthesis, which would naturally be required for cell proliferation. In support of this being a factor in axolotl heart regeneration, we found indicative evidence for increased biosynthesis of both purines and pyrimidines in our metabolomics data when comparing heart samples at baseline vs. 4 dpi (Figure 6). Ribose‐5‐phosphate is a required substrate for nucleotide biosynthesis and comes from the conversion of glucose‐6‐phosphate by the pentose phosphate pathway. In line with this, we also showed an increased activity of G6PD (which performs the initial step of converting glucose‐6‐phosphate to 6‐phosphogluconolactone) in and around the injured area throughout the regenerative period with enzyme histochemistry (Figure 9B,E). While increased activity of G6PD may at first seem incompatible with the low uptake of ^18F^‐FDG in the same areas (Figure 4), it is important to realize that the ^18F^‐FDG signal is not zero within the injury but simply comparatively low to the uninjured myocardium. It is possible that the uninjured myocardium takes up a greater amount of glucose which is then almost exclusively utilized for aerobic glycolysis, while other areas like the injury border zone take up a comparatively smaller amount of glucose, but then shuttles it preferably toward the pentose phosphate pathway. Signal from within the injury itself could also be coming from immune cells that infiltrate the area after injury.

G6PD activity was also prominent in the epicardium, both at baseline and during regeneration (Figure 9B,E). Previous research has shown that the epicardium represents a key compartment of proliferative cells in regenerative hearts.^8^, ^9^, ^72^ It is possible that a different metabolic phenotype, for instance, involving increased reliance on the pentose phosphate pathway, could be an important factor in priming epicardial cells for proliferation in response to injury. G6PD, importantly, also helps maintain reduced glutathione.^73^ An increased myocardial uptake of ^14^C‐acetate peaked at 30 dpi, especially within the border zone area (Figure 4B,F–I). Acetate can be shuttled into both OXPHOS and biosynthesis from the TCA cycle. It seems plausible that acetate would be necessary to facilitate the biosynthesis of lipids required for building new cell membranes.

Overall, metabolic activity was dramatically different within the uninjured myocardium vs. the remaining injury. On autoradiography, the injury itself compared to the uninjured myocardium displayed a comparatively low uptake of both ^18^F‐FDG and ^14^C‐acetate (Figure 4) and a low level of SDH activity (Figure 9B). The specific roles of SDH activity in the context of regeneration may be of particular interest in the future. In zebrafish, it has been demonstrated that SDH activity was 40% reduced in border‐zone CM at 7 days after cardiac cryo‐injury compared to the more remote CMs.^74^ Furthermore, it has been shown in neonatal mice that inhibition of SDH could promote heart regeneration beyond the typical window of regenerative capacity during the first few days after birth.^75^

Studies in human cancer cells have demonstrated that the loss of SDH is linked to the accumulation of succinate, leading to increased ROS, breakdown of the TCA cycle, and increased reliance on glycolysis.^76^ Alternative mechanisms involving succinate could be of key importance to how axolotl CMs both manage oxidative stress and maintain a proliferative phenotype while increasing OXPHOS. Species‐specific adaptive mechanisms involving succinate metabolism during hypoxia have, for instance, been documented in some species of turtles to cope with sudden and dramatic rises in oxygen exposure while avoiding reperfusion injury.^77^

We also found an upregulation of mTOR activity (Figure 8A–E), specifically in and around the injury site at 4 and 14 dpi, which is one of the possible key signaling pathways pushing cardiac metabolism toward a regenerative phenotype.^26^ There are some confounding reports on the role of mTOR in cardiac regeneration. While some have reported mTOR as being required for cardiac regeneration in neonatal mice and zebrafish,^26^ others have demonstrated mTOR to be downregulated in zebrafish 3 days after cardiac resection injury.^78^ Our findings prompt further questions about the interaction of hypoxia/hyperoxia and mTOR in the axolotl. Barna et al. reported limb regeneration in axolotls to be associated with a rapid uptick in protein synthesis, regulated by mTOR.^79^ They also found that axolotls expressed a unique sequence expansion in the mTOR gene, providing a more easily activated complex that they state could convey a heightened ability for axolotl mTOR to respond to nutrient changes and induce a rapid metabolic response to injury.^79^ How this relationship plays out in the context of heart regeneration or if it may have any link to the robustness of axolotl heart regeneration in hyperoxic conditions warrants further study.

In conclusion, our results demonstrate that axolotl heart regeneration involved a wide range of metabolic changes that might be required to different degrees for the heart to repair itself after cryo‐injury. While this explorative study cannot conclude which of these observed metabolic effects are driving the regenerative process, we saw a dynamic upregulation of glucose uptake, acetate uptake, and nucleotide biosynthesis pathways at different time points in line with previous studies in other species, suggesting that some of these processes are fundamental and shared among regenerative species. We also detected several interesting metabolites with possible links to regeneration and found that axolotl heart regeneration was unaffected by hyperoxia, and thus, axolotls might possess unique protective mechanisms against ROS not found in mice or zebrafish.

## 4 EXPERIMENTAL PROCEDURES

### 4.1 Animal procedures

Axolotls were housed individually in plastic crates at 20°C and fed 4–5 axolotl pellets three times weekly (Axobalance, Aquaterratec Germany) throughout the experiment (24 h since last meal at each measurement time point). Prior to echocardiography, blood sampling, and surgery, animals were anesthetized by submersion in 0.2% benzocaine for 30 min. Cryo‐injury was performed as previously described.^43^ Briefly, the ventricle was exposed, and a localized cryo‐injury was induced by placing a copper probe cooled in liquid nitrogen on the ventroapical face for 10 s. The probe was released by applying sterile amphibian Ringer's solution, and the incision was closed using 4–5 individual sutures. A total of 24 axolotls were utilized to collect ventricles in cryo‐embedding medium at different time points for autoradiography, histology, and immunofluorescence. The animals in the later time point groups were used for respirometry as well as blood metabolite assays to obtain paired data. In addition, 20 animals harvested at different time points were used to collect ventricles for homogenization and subsequent metabolomics analysis. Finally, 21 animals were used for the hyperoxia experiments. All animals in the study were adults in a size range of 37–68 g body mass and 12–20 cm in snout‐to‐tail length acquired from a commercial breeder in Denmark (Table 2). All animal experiments were approved by the Danish animal experiments inspectorate under approval number 2020‐15‐0201‐00688.

**TABLE 2:** Animal information.

Experiment/batch	*n*	Age at experiment start	Body mass
Main experiment: respirometry, blood sampling, echocardiography, autoradiography, immunofluorescence, and enzyme histochemistry. A more detailed description of data collected from each animal is given in Supplementary Table 3.	24	20 months (1 year 8 months)	55.08 ± 8.62 g
Hyperoxia experiments	21	Unknown	48.29 ± 5.74 g
Metabolomics	20	16 months (1 year, 4 months)	42.36 ± 4.02 g

### 4.2 Echocardiography

Anesthetized animals were placed in a supine position and covered in water just above the chest area. Imaging and subsequent analysis were performed as previously described using the Visualsonics Vevo 2100 ultrasound system.^80^ Long‐axis views were obtained in B‐mode along with doppler measurements from the main outflow tract. The Vevo Lab Software was used to determine heart rate, ventricular area at end‐diastole and end‐systole, as well as non‐contracting area at the maximum size of the non‐contracting area. All measurements were obtained as an average across three cardiac cycles. Calculations were applied as previously described.^40^ In brief, heart rate is determined as the time between peaks on Doppler measurements. The volume of the ventricle and non‐contracting area was determined by assuming a spherical ventricular shape and a hemispherical non‐contracting area shape to calculate volume from area tracings. VFAC was then calculated as the % change between end‐diastolic volume and end‐systolic volume. Cardiac output was calculated as the end‐diastolic volume minus the end‐systolic volume divided by heart rate and normalized to the body mass of the animals. For measurements pertaining to cardiac function (Figure 1A–C), we opted to evaluate the 120 dpi subgroup in the main experimental group (Table 2) as these were the only animals available for a full time series and repeated measures statistics. For evaluation of non‐contracting fraction, we intended to include all animals from the main experiment (not including the baseline group that was not injured); however, the 4 and 60 dpi subgroups could not be evaluated at the correct times due to a technical problem with the ultrasound system. Thus, the 14, 30, and 120 dpi subgroups were included in the analysis (a detailed overview is presented in Supplementary Table 3).

### 4.3 Respirometry

Axolotls were gently placed in a respirometry chamber (1 L glass container with air‐tight lid). The container was filled completely with aerated water (20.95% O_2_) and all air bubbles were removed by filling the container through a narrow filling hole using a 50 mL syringe before covering the filling hole with Leukoplast Sleek tape (water‐tight medical grade tape). Axolotls were left undisturbed for 2 h, and the oxygen content of the water was measured in a 30 mL water sample at the start and end of the time spent in the chamber using a Unisense OXY‐meter. The water sample obtained at the end of the experiment was collected by inserting a syringe through the tape. Before collecting the end sample, the container was gently inverted to prompt the animals to move around and create mixing in the water. Respirometry was performed in a temperature‐controlled room at 20°C, and the water temperature was also measured in the samples using a temperature probe. In addition, relative humidity and barometric pressure were noted from sensors in the room. The animals were last fed 24 h prior to each measurement. Oxygen content in the sample was ultimately calculated as: ΔO_2_ × time^−1^ × body mass ^−1^ and adjusted for water mass, temperature, barometric pressure, relative humidity, and vapor pressure.

### 4.4 Plasma lactate measurements

Blood samples of approximately 100–150 μL were collected just prior to heart harvest after ∼24 h of fasting from a major gill vein using a 0.5 mL/30 G heparinized syringe. After collection, samples were centrifuged at 2000*g* for 5 min, plasma collected, snap frozen, and stored at −80°C. Prior to the microplate assay, samples were deproteinized using a commercial kit (Abcam; Cat.nr: ab204708). Lactate was measured in 5 μL samples diluted to a total volume of 100 μL using a commercial kit (Sigma Aldrich; Cat.nr: MAK065). All assays were performed according to the respective manufacturers' protocols, included a standard, and assays were analyzed on a Biochrome ELISA plate reader.

### 4.5 Hyperoxia treatments

To investigate the effect of increased oxygen levels on the capacity of the axolotl heart to regenerate after cryo‐injury, we used two approaches: A constantly elevated O_2_ level (constant normobaric hyperoxia) by continuously bubbling housing water with pure molecular O_2_ at normobaric conditions and hyperbaric oxygen treatment (HBOT) where animals were exposed to O_2_ at three atmospheres absolute (303.9 kPa) for 2 h daily for the first 10 days of the regenerative process and then 5 days per week for the remaining 60 days. During HBOT, O_2_ was likewise delivered by bubbling housing water to ensure complete O_2_ saturation. We tested if the two treatments led to an increase in intracardiac plasma pO_2_ in sham and cryo‐injured axolotls at 4 dpi using a calibrated O_2_ microsensor (tip diameter: 100 μm) (Unisense, Denmark) surgically inserted into the ventricular lumen. Additionally, we measured intracardiac hemoglobin saturation at 4 dpi in hyperoxia‐treated sham and cryo‐injured axolotls using photoacoustics imaging (VisualSonics Vevo 2100 LAZR). Thereafter, in two separate experiments, we tested the regenerative capacity of cryo‐injured hearts receiving either normoxia vs. normobaric constant hyperoxia or normoxia vs. HBOT treatment using echocardiography at day 0, 4, 7, 14, 21, 30, 40, 51, and 70 post injury, as described above, and quantitative histology upon termination at day 73 post injury to measure infarction fraction.

### 4.6 Autoradiography

Axolotls were anesthetized and administered a bolus injection in the jugular vein consisting of a high dose of ^18^F‐FDG (∼20 MBq) and a much smaller dose of ^14^C‐acetate (200 kBq) dissolved in saline. After injection, the animals were allowed to wake up for 2 h before they were re‐anesthetized and the heart harvested 30 min later, following an injection of 50 μL 500 IU heparin. The heart was gently rinsed free of blood and frozen in the vapor phase of liquid nitrogen and embedded in M‐Freeze (Sigma Aldrich; Cat.nr: 103693). As an internal reference, a piece of liver tissue was simultaneously collected and used for normalization of heart autoradiogram signal intensities. Immediately after harvest, the liver sample was sectioned with three sections from each animal at 10 μm thickness. The heart was then sectioned at 10 μm thickness and every 16th section was collected for autoradiography and subsequent histology sampling the entire ventricle from start to end. Additional sections were harvested and stored at −80°C for immunofluorescence and enzyme activity stains.

The autoradiography sections were developed and imaged at two separate times. First, immediately after sectioning, the signal was read by a brief ~1‐h exposure of tissue sections to a phosphor imaging plate. Due to the administration of a much higher dose of ^18^F‐FDG than ^14^C‐acetate, this first obtained autoradiogram overwhelmingly reflects the signal from ^18^F‐FDG. A second round of exposure to phosphor imaging plates was conducted about a week later when no ^18^F‐signal remained due to the short half‐life of 110 min^81^ (compared to 5730 years for ^14^C‐signal^82^). To compensate for the low dose of ^14^C‐acetate, the exposure time for this second round of autoradiography was prolonged to ~5 days while using lead shielding to minimize background contamination. Plates were read at a resolution of 25 μm using an Amersham Typhoon Biomolecular imager (GE Healthcare, Marlborough, USA). Autoradiography images were analyzed using ImageJ.^83^ Briefly, three sections in which both uninjured and injured tissue were evident were chosen from each animal. The signal intensity was normalized by first subtracting the signal value of the background where no sample was present and then divided by the mean signal intensity of the liver sections from the same animal to derive a normalized photo‐stimulated luminescence (normalized PSL) value. An ROI was drawn around the uninjured myocardium, the infarction area, as well as the combined area. From these ROIs, the mean, maximum, and minimum signal intensity were obtained and averaged across the three sections. In the case of ^14^C‐acetate, a separate analysis was performed on the same three sections from each animal to evaluate the dependency of signal intensity and proximity to the injury. In imageJ,^84^ the plugin “Concentric circles” was used, with the center of the infarction set as the center of the innermost circle. This divided the section into 4 zones at varying distances from the infarction. A grid was then superimposed, and the signal value at each intersection point was obtained along with its respective zone.

### 4.7 LC–MS Untargeted Metabolomics Analysis

Frozen tissue samples were processed into dry extracts by precipitation in cold 80% methanol and reconstituted in water with 0.1% formic acid. Samples were analyzed on an ACQUITY I‐Class UPLC system (Waters Corporation, Milford, MA, USA) equipped with an ACQUITY UPLC HSS T3 C18 column (2.1 mm × 100 mm, 1.8 μm, Waters) and coupled to a Bruker maXis Impact QTOF mass spectrometer (Bruker Daltonics, Bremen, Germany). The QTOF‐MS instrument was operated in both positive and negative electrospray ionization (ESI) mode. Metabolites were confirmed to be identified when the *m/z* value, retention time, and fragments corresponded with an authentic standard compound. Tentative annotations were based on *m/z* values and fragments corresponding with external databases. In cases of features with no clear fragmentation patterns, annotations were additionally made if the *m/z* value and retention time corresponded to an authentic standard compound in our in‐house database. Tentative annotations based solely on *m/z* values were not included. Statistical analysis was performed by using an unpaired *t*‐test between baseline samples and other time points. A more detailed description of the methods involved in metabolomics processing and analysis is provided in Supplementary Methods.

### 4.8 Histology and Immunofluorescence

The same sections used for autoradiography were later stained with Masson's trichrome stain according to the manufacturer's protocol (Abcam; Cat.nr: ab150686) except for Bouin fixation being carried out at room temperature overnight rather than in a warm water bath due to the sensitivity of cryosections. Furthermore, the sections were pre‐fixed in cold acetone for 10 min after 5 min of thawing. Stereology was performed using 15–25 sections from each heart (depending on ventricle size) in QuPath^85^ as previously described.^86^, ^87^ In brief, a 500 μm × 500 μm point grid was superimposed on the images, and for each point within the section, a designation of “myocardium” or “infarction” was given, and the infarction fraction was calculated as the percentage of the total number of points designated as infarction area. The uninjured vs. injured area was recognized at all stages due to abnormal morphology and/or deposition of collagenous material, which stains blue, while muscle, red blood cells, and cytoplasm stain red.

For immunohistochemistry, a general protocol was followed in which sections were initially thawed for 5 min prior to fixation in cold 4% paraformaldehyde, incubated 15 min in permeabilization buffer (0.25% Tritron‐X), and 3 h in blocking buffer (5% goat serum, 3% BSA, and 0.1% Tritron‐X). Primary antibodies were incubated overnight at 4°C and then secondary antibodies at room temperature for 2 h. Finally, sections were mounted with Invitrogen Prolong Diamond antifade mounting media with DAPI (Thermo Fisher; Cat.nr: 17137388). Negative controls were done using the same concentration of secondary antibody without primary antibody, as well as using an IgG Rabbit isotype control, both of which yielded no visible signal within microscope settings used during image acquisition. All reagents and specific antibodies are listed in Supplementary Table 4. All sections were imaged using an Olympus VS120 slide scanner, and immunofluorescence images were analyzed using QuPath^85^ to measure the percentage positive area using the thresholder function on three sections within the infarcted area per animal.

### 4.9 Enzyme Histochemistry

Enzyme histochemistry was performed according to the methods described by Van Noorden and Frederisk.^88^ In brief, unfixed cryosections were incubated in a reaction mix consisting of polyvinyl alcohol in a 0.1 M Tris‐maleate or Tris–HCL buffer containing sodium azide, nitroblue tetrazolium chloride, and either methylenediphenazine methosulfate or phenazine methosulfate (to prompt a tetrazolium salt reaction), as well as the appropriate substrates (d‐glucose 6‐phosphate, NADP^+^ and MgCl for G6P, sodium lactate and NAD^+^ for LDH, and sodium succinate for SDH). After incubating for 10–20 min, purple formazan was precipitated in areas with enzyme activity. The sections were washed and fixed for 5 min in 4% PFA before mounting with Eukitt mounting media. An extensive list of reagents is given in Supplementary Table 3. Incubation with the reaction mix alone (without substrates) did not result in any formazan precipitation. An illustration showing the principle of the tetrazolium salt to formazan reaction is shown in Supplementary Figure 6.

## FUNDING INFORMATION

The Lundbeck Foundation (R324‐2019‐1470), The A.P. Møller Foundation (19‐L‐0275), and the Carlsberg Foundation (CF21‐0605).

## CONFLICT OF INTEREST STATEMENT

The authors declare no competing interests. No funding body had any influence on the design of the study or the presentation of the results.

## Supporting information


**Supplementary Figure 1.** Echocardiography Analysis. Representative screenshots of echocardiography analysis after cryo‐infarction. Blue lines indicate tracings. V = ventricle; I = infarction (non‐contacting area); A = atria; OT = outflow tract. All images (and videos) are from the same animal. Corresponding video files allowing visualization of the non‐contracting area at 4, 14, 30, and 60 dpi and baseline are available in Supplementary Video 1.

**Supplementary Figure 2.** Masson's Trichrome staining of paraffin embedded axolotl ventricles after cryo‐infarction. dps = days post sham; dpi = days post injury. Ventricles were fixated in formalin and embedded in paraffin for sectioning. Note that at 94 dpi, the remaining scar tissue is in the process of being expelled.

**Supplementary Figure 3.** Corresponding brightfield images for Figure 4 (autoradiography). Masson's trichrome stain of sections used for autoradiography in Figure 4. Two different sections were used for the 14 dpi time point and for 30 dpi a neighboring section is shown here because the section shown in Figure 4 was lost during post‐fixation prior to staining. Baseline hearts were not stained for Masson's Trichrome.

**Supplementary Figure 4.** Analysis of 14C‐acetate signal according to proximity to infarction.

**Supplementary Figure 5.** Neighboring brightfield images for Figure 8 (Immunofluorescence). Masson's trichrome stain of sections neighboring those used for autoradiography in Figure 8. Baseline hearts were not stained for Masson's Trichrome.

**Supplementary Figure 6.** Tetrazolium salt to formazan reaction. The tetrazolium salt method can be used to demonstrate the activity of any dehydrogenase provided the correct substrate and cofactors are added to the staining solution. Tetrazolium salts are initially yellow or colorless and fairly soluble in water but can be reduced to water‐insoluble formazan crystals which have a bright purple color, which can be easily seen on tissue sections. Because the redox potential in transferring electrons from the involved coenzymes to tetrazolium salts to produce formazan is not strong enough, the electrons can become captured and instead reduced via OXPHOS, and so to ensure a specific signal, an endogenous electron carrier like phenazine methosulfate or methoxyphenazine methosulfate must be added to the staining solution to speed up the reaction. As an example, here the reaction of lactate dehydrogenase is illustrated in the presence of a tetrazolium salt‐containing reaction mix. The reaction mix is prepared in polyvinyl alcohol to avoid any diffusion of oxygen from the environment.

**Data S1.** Supporting Information.

**Video S1.** Echocardiography of the regenerating axolotl heart. B‐mode echocardiography at the maximum ventricular size in diastole from the same animal pre‐injury, and at 4, 14, 30, and 60 dpi. Time point is written in top right corner. The injury is identified as the non‐contracting area in the top left edge of the ventricle.

## Data Availability

The data that support the findings of this study are available from the corresponding author upon reasonable request.
