# Supplementary figures and images for: Metabolic changes during cardiac regeneration in the axolotl
Source: Dev Dyn. 2025 Mar 22;255(1):66–85. doi: 10.1002/dvdy.70020 (PMC12862128; doi:10.1002/dvdy.70020)

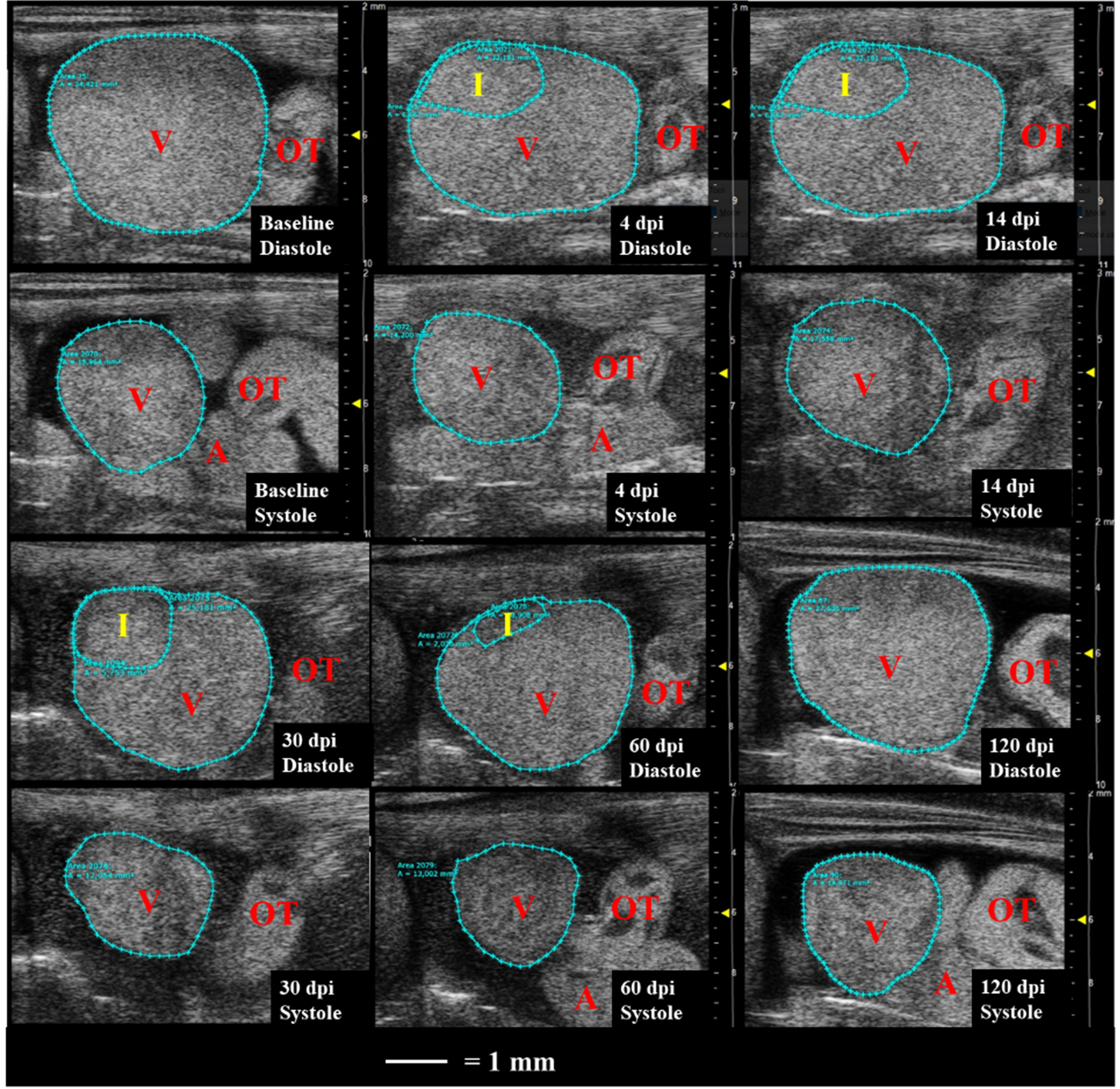

Supplement: Supplementary file 1 — Supplementary Figure 1. Echocardiography Analysis. Representative screenshots of echocardiography analysis after cryo‐infarction. Blue lines indicate tracings. V = ventricle; I = infarction (non‐contacting area); A = atria; OT = outflow tract. All images (and videos) are from the same animal. Corresponding video files allowing visualization of the non‐contracting area at 4, 14, 30, and 60 dpi and baseline are available in Supplementary Video 1. [file DVDY-255-66-s001.tif]

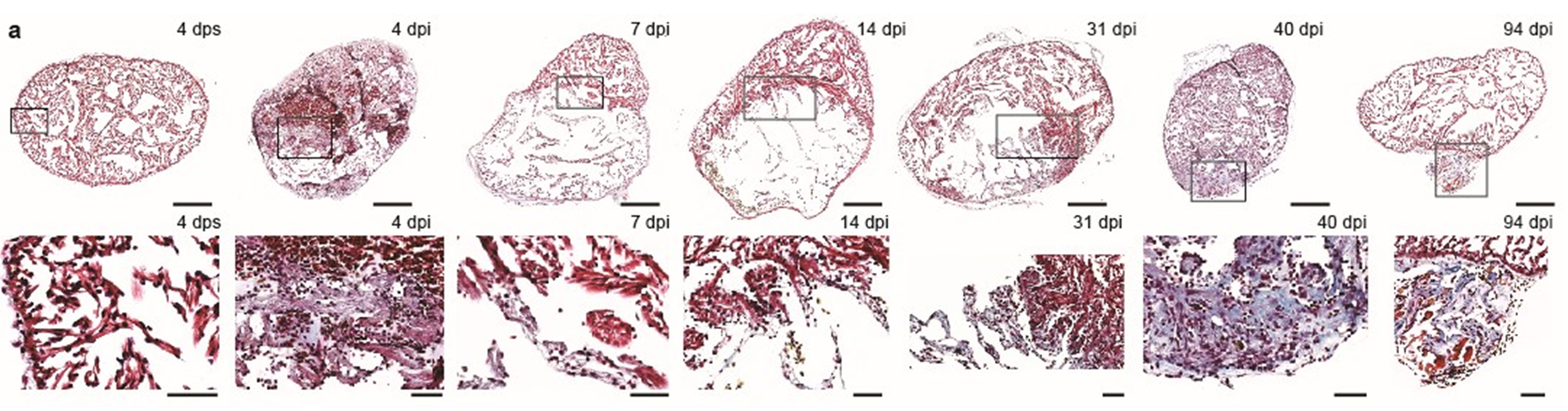

Supplement: Supplementary file 2 — Supplementary Figure 2. Masson's Trichrome staining of paraffin embedded axolotl ventricles after cryo‐infarction. dps = days post sham; dpi = days post injury. Ventricles were fixated in formalin and embedded in paraffin for sectioning. Note that at 94 dpi, the remaining scar tissue is in the process of being expelled. [file DVDY-255-66-s003.tif]

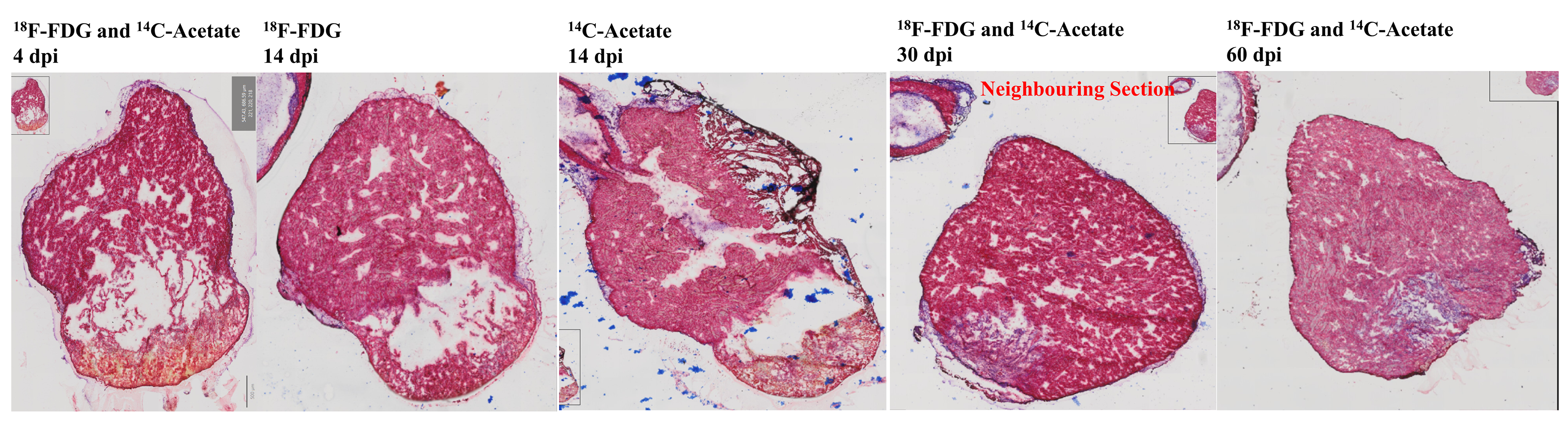

Supplement: Supplementary file 3 — Supplementary Figure 3. Corresponding brightfield images for Figure 4 (autoradiography). Masson's trichrome stain of sections used for autoradiography in Figure 4. Two different sections were used for the 14 dpi time point and for 30 dpi a neighboring section is shown here because the section shown in Figure 4 was lost during post‐fixation prior to staining. Baseline hearts were not stained for Masson's Trichrome. [file DVDY-255-66-s004.tif]

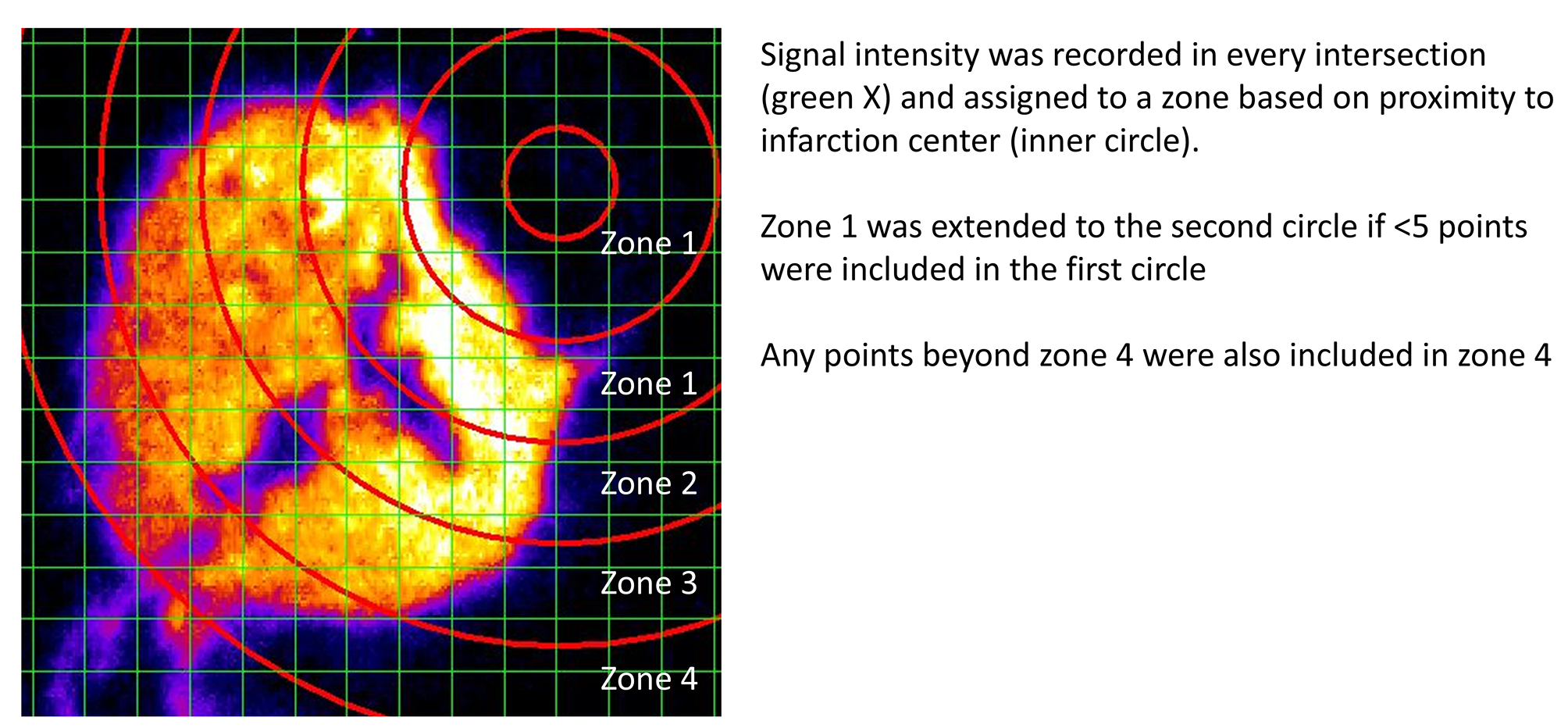

Supplement: Supplementary file 4 — Supplementary Figure 4. Analysis of 14C‐acetate signal according to proximity to infarction. [file DVDY-255-66-s006.tif]

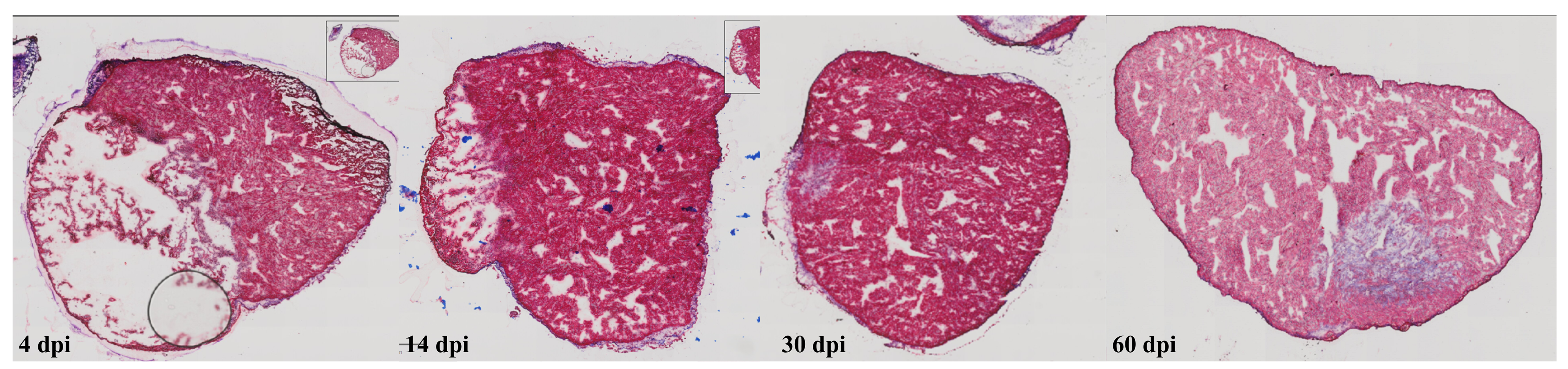

Supplement: Supplementary file 5 — Supplementary Figure 5. Neighboring brightfield images for Figure 8 (Immunofluorescence). Masson's trichrome stain of sections neighboring those used for autoradiography in Figure 8. Baseline hearts were not stained for Masson's Trichrome. [file DVDY-255-66-s007.tif]

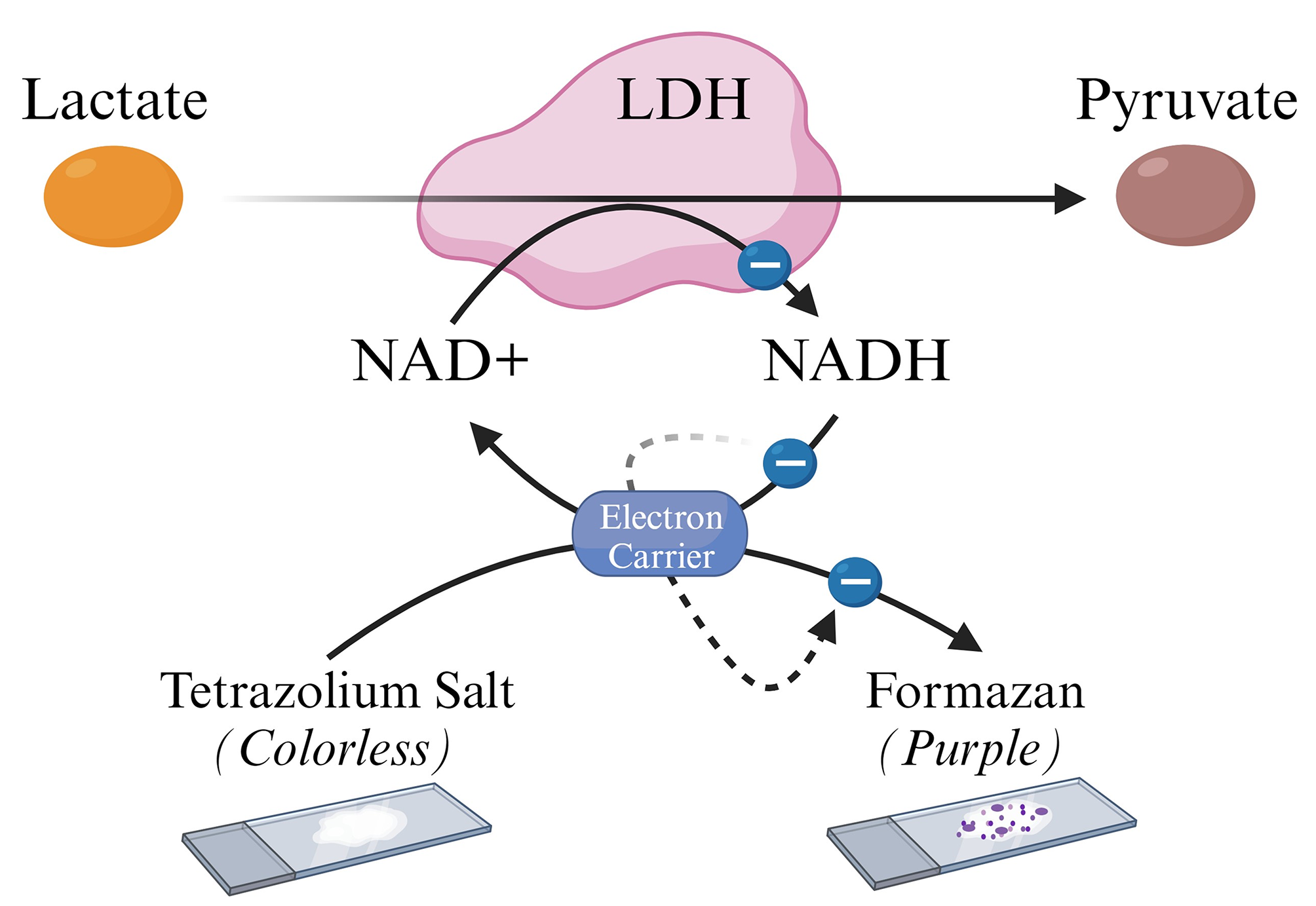

Supplement: Supplementary file 6 — Supplementary Figure 6. Tetrazolium salt to formazan reaction. The tetrazolium salt method can be used to demonstrate the activity of any dehydrogenase provided the correct substrate and cofactors are added to the staining solution. Tetrazolium salts are initially yellow or colorless and fairly soluble in water but can be reduced to water‐insoluble formazan crystals which have a bright purple color, which can be easily seen on tissue sections. Because the redox potential in transferring electrons from the involved coenzymes to tetrazolium salts to produce formazan is not strong enough, the electrons can become captured and instead reduced via OXPHOS, and so to ensure a specific signal, an endogenous electron carrier like phenazine methosulfate or methoxyphenazine methosulfate must be added to the staining solution to speed up the reaction. As an example, here the reaction of lactate dehydrogenase is illustrated in the presence of a tetrazolium salt‐containing reaction mix. The reaction mix is prepared in polyvinyl alcohol to avoid any diffusion of oxygen from the environment. [file DVDY-255-66-s005.tif]
