# Supplementary material for: Metabolic changes during cardiac regeneration in the axolotl
Source: Dev Dyn. 2025 Mar 22;255(1):66–85. doi: 10.1002/dvdy.70020 (PMC12862128; doi:10.1002/dvdy.70020)
Supplement: Supplementary file 7 — Data S1. Supporting Information. [file DVDY-255-66-s002.docx]

**SUPPLEMENTARY**

**Supplementary Methods**

**LC-MS Untargeted Metabolomics Analysis**

***Extraction of Metabolites from Heart Tissue***

Ventricular tissue samples ∼30 mg was maintained at -80 °C until extraction and analysis. Frozen tissue was allowed to thaw on ice before homogenization in 1 mL ice-cold 80% methanol in a Precellys Evolution tissue homogenizer (Bertin Technologies, Montigny-le-Bretonneux, France) (5800 rpm: 15 sec mix, 30 sec pause, 15 sec mix, 5 °C) using a Precellys Lysing Kit (Soft tissue homogenization CK14, Bertin Technologies, Montigny-le-Bretonneux, France). Samples were allowed to incubate for 10 min on ice before centrifugation (10,000 *g* for 10 min at room temperature). The supernatant (700 µL) was collected and transferred to a glass tube and placed on ice. The pellet was added 0.5 ml ice-cold 80% methanol and homogenization, incubation and centrifugation were repeated. The supernatant (400 µL) was transferred to the same glass tube as before and evaporated to dryness in a SpeedVac at 40 °C. The dried extracts were reconstituted in 100 µL/ 7 mg tissue of 0.1% formic acid in water. A quality control (QC) sample was prepared by pooling equal volumes of all samples.

***UPLC-HR-QTOF-MS Analysis***

All samples were analyzed on an ACQUITY I-Class UPLC system (Waters Corporation, Milford, MA, USA) equipped with an ACQUITY UPLC HSS T3 C18 column (2.1 mm × 100 mm, 1.8 µm, Waters) and coupled to a Bruker maXis Impact QTOF mass spectrometer (Bruker Daltonics, Bremen, Germany).

Mobile phase A consisted of 0.1% formic acid (v/v) in water and mobile phase B consisted of 0.1% formic acid (v/v) in methanol/acetonitrile (1:1 v/v). The flow rate was 0.4 mL/min at a column temperature of 50 °C. The gradient was as follows: 0% B (0-2 min), 0-40% B (2-6 min), 40-60% B (6-6.5 min), 60-80% B (6.5-11 min), 88-100% B (11-11.5 min), 100% B (11.5-18 min), 100-0% B (18-19.5 min) and 0% B (19.5-21 min). The sample were kept in the autosampler at 6 °C and the injection volume was 5 µL.

The QTOF-MS instrument was operated in both positive and negative electrospray ionization (ESI) mode with a capillary voltage of 4000 V in positive ESI and 2500 V in negative ESI. The nebulizing gas pressure was 1.2 bar, and the drying gas flow and temperature were 11.0 L/min and 220 °C. A mass range of 50-1000 *m/z* and a sampling rate of 4Hz were used for MS-analysis. Auto-MS/MS (data-dependent acquisition) with a collision energy of 20 eV and a sampling rate of 10 Hz were used for fragmentation of molecular features for annotation and identification.

Internal calibration was performed at the end of each run using sodium formate in both positive and negative ESI. Four QC samples were injected in the beginning of a batch before sample analysis. Samples were injected randomly with a QC sample between every 5th sample for quality assessment of instrument performance within each batch.

***Data Preprocessing***

The raw UPLC-HR-QTOF-MS data was converted to *mzML*. file format using MSConvert (ProteoWizard, Palo Alto, CA, USA). The *mzML*. files were processed with *XCMS*^S1^ (version 3.12.0) in R (version 4.0.3). The *CentWawe* algorithm^S2^ was used for peak picking with a resolution of 12 ppm and a signal-to-noise threshold (snthr) set to 6. The *Obiwarp* algorithm^S3^ was used for retention time correction. Gap filling was conducted to recover missing signals in the raw data. Only features present in more than 70% of the samples within one sample group and with a coefficient of variation (CV) < 30% between the QC samples were considered for further analysis. Isotopes, adducts and ion source fragments were annotated using *CAMERA*^S4^ (version 1.46). Each sample was normalized to the total peak intensity. A tabulated data matrix with aligned retention times and *m/z*-values was summarized in a spreadsheet and used for data analysis.

***Metabolomics Data Analysis***

Principal component analysis (PCA) was performed in SIMCA (version 16.0.1, Sartorius Stedim Data Analytics, Goettingen, Germany). PCA was used to show potential clustering trends of the sample groups and the QC-samples. UV scaling was applied before PCA.

An unpaired two-sample *t*-test was used to examine possible differences between baseline and other time points. False discovery rate (FDR) *q*-values based on the Benjamini and Hochberg method^S5^ were calculated to account for false discoveries.

***Metabolite Annotation and Identification***

Automatic annotation of features was performed using *metid^S6^* (version 1.2.24) in R (version 4.2.1). Data was compared with our in-house database containing 549 compounds as well as external public databases from MassBank, NIST, HMDB (https://mona.fiehnlab.ucdavis.edu) and those available in the *metid* package. Selected features were additionally searched based on *in silico* fragmentation searches in MetFrag (<https://msbi.ipb-halle.de>) based on the KEGG database (<https://www.genome.jp/kegg/>).

Metabolites were confirmed identified when the *m/z*-value, retention time and fragments corresponded with an authentic standard compound. Tentative annotations were based on *m/z*-values and fragments corresponding with external databases. In cases of features with no clear fragmentation patterns, annotations were additionally made if the *m/z*-value and retention time corresponded to an authentic standard compound in our in-house database. Tentative annotations based solely on *m/z*-values were not included. All metabolites selected as interesting were sought to be fully identified.

**Supplementary Data and Results**

**Supplementary Table 1. Studies of intrinsic heart regeneration in salamanders.** Relevant studies listed in order of publication year.

| **Species** | **Keywords** | **Main findings** | **Injury model** | **Reference** |
| --- | --- | --- | --- | --- |
| Newt (*Notophthalmus viridescens*) | Regeneration, Proliferation | Response to injury involved blood clot formation, coagulation necrosis, macrophage activity, regenerative activity and connective tissue formation. DNA synthesis and mitosis in CMs was shown using electron microscopy. | Resection-injury | *Response of the adult newt ventricle to injury (1974)*^6^ |
| Newt *(Notophthalmus viridescens)* | Regeneration, Proliferation | When a resected piece of heart tissue was minced and placed back onto the resection surface, cells within the minced tissue could proliferate and synchronize its contraction with the injured heart but could not fully reintegrate anatomically. | Resection injury | *Repair and reorganization of minced cardiac muscle in the adult newt (Notophthalmus viridescens) (1978)*^90^ |
| Newt *(Notophthalmus viridescens)* | Proliferation | Atrial cells proliferated after injury shown by thymidine incorporation and mitosis identified by electron microscopy. | Resection-injury | *The atrial proliferative response following partial ventricular amputation in the heart of the adult newt. A light and electron microscopic autoradiographic study (1983)*^91^ |
| Axolotl (*Ambystoma mexicanum*) | Proliferation | After injury, cells in the atria and ventricle underwent DNA synthesis shown by brdU incorporation in CM-specific marker positive cells. The newly formed epicardium was also brdU positive. No brdU positive cells were seen in the myocardium or epicardium of sham operated animals. | Resection-injury | *Cell cycle reentry of ventricular and atrial cardiomyocytes and cells within the epicardium following amputation of the ventricular apex in the axolotl, Amblystoma mexicanum: Confocal microscopic immunofluorescent image analysis of bromodeoxyuridine-labeled nuclei (2002)*^8^ |
| Newt *(Notophthalmus viridescens)* | Proliferation | Newt CMs in culture displayed different abilities for proliferation with only about one third successfully completing mitosis, with an ability for daughter cells to also proliferate. Thus, only a subset of newt CMs likely proliferate and contribute to regeneration. | *In Vitro* | *Heterogeneous proliferative potential in regenerative adult newt cardiomyocytes (2003)*^92^ |
| Axolotl (*Ambystoma mexicanum*) | Regeneration | After injury, the area was first filled in by a blood clot and collagen fibers and then muscle. Bis-Benzimide assay and co-staining with PCNA and alpha-sarcomeric actin indicated proliferation in the injured area. | Resection-injury | *Myocardial regeneration in Ambystoma mexicanum after surgical injury (2005)*^93^ |
| Newt  *(Notophthalmus viridescens)* | Dedifferentiation | After injury CMs dedifferentiated as evident by reduced expression of sarcomeric proteins. CMs implanted into limb could transdifferentiate to skeletal muscle or chondrocyte lineages via contact with the limb injury blastema as CMs implanted in intact limbs retained CM identity. | Crush-injury | *Re-programming of newt cardiomyocytes is induced by tissue regeneration (2006)*^94^ |
| Axolotl (*Ambystoma mexicanum*) | Heart function, Proliferation | After injury proliferating cells were detected in the heart and heart function was gradually restored over 90 days | Resection-injury | *Functional and structural regeneration in the axolotl heart (Ambystoma mexicanum) after partial ventricular amputation (2010)*^95^ |
| Newt *(Notophthalmus viridescens)* | Gene expression | Comparing gene expression in newt and zebrafish identified similarities in gene regulation of transcripts associated with the GO-terms: Cell cycle, cell migration, and proliferation in response to injury | Crush-injury | *Analysis of newly established EST databases reveals similarities between heart regeneration in newt and fish (2010)*^96^ |
| Newt *(Notophthalmus viridescens)* | Regeneration, Proliferation | Newts regenerated after injury without permanent scarring. Increased proliferation was shown along with expression of genes associated with cardiac development like Islet1 and GATA4. | Resection-injury | *Recapitulation of developmental cardiogenesis governs the morphological and functional regeneration of adult newt hearts following injury (2011)*^97^ |
| Newt *(Notophthalmus viridescens)* | ECM | Damaged CMs died by necrosis and were removed in the first week after injury before new thin trabecula filled in and replaced the damaged area. Deposition of ECM containing collagen III directed the outgrowth of forming trabecula containing CMs | Crush-injury | *Reconstitution of the myocardium in regenerating newt hearts is preceded by transient deposition of extracellular matrix components (2013)*^98^ |
| Newt (Notophthalmus viridescens) | ECM | Migration and proliferation of epicardial cells contributed to heart regeneration, facilitated by specific pro-regenerative ECM components including tenascin-C, hyaluronic acid and fibronectin | Resection-injury | *A Dynamic Spatiotemporal Extracellular Matrix Facilitates Epicardial-Mediated Vertebrate Heart Regeneration (2013)*^99^ |
| Newt (Notophthalmus viridescens) | miRNA, ECM | mir-128 was upregulated during proliferation of CMs and non-CMs in the injury border zone during heart regeneration. mir-128 was involved in ECM deposition and activation of Islet1. | Resection-injury | *miR-128 regulates non-myocyte hyperplasia, deposition of extracellular matrix and Islet1 expression during newt cardiac regeneration (2013)*^100^ |
| Axolotl (*Ambystoma mexicanum*) | Epigenetics | Baf60c, a component of ATP-dependent chromatin-remodeling complexes was upregulated after injury in the axolotl and temporally associated with CM proliferation. Knockdown of the same factor reduced proliferation of CMs in neonatal mice. | Resection-injury | *Expression analysis of Baf60c during heart regeneration in axolotls and neonatal mice (2016)*^101^ |
| Axolotl *(Ambystoma mexicanum)* | Immune response, ECM | Depletion of macrophages during early time point after injury led to regenerative failure. Instead of transient scarring animals had permanent scar after injury due to alternative fibroblast activation and lysyl-oxidase enzyme synthesis. | Cryoinjury | *Heart regeneration in the salamander relies on macrophage-mediated control of fibroblast activation and the extracellular landscape (2017)*^42^*.* |
| Axolotl (*Ambystoma mexicanum*) | Immune response | Complement receptors were upregulated across three regenerative species after injury (axolotl, zebrafish and neonatal mice). Inhibition of complement 5a receptor 1 reduced cardiomyocyte proliferation after injury in all three species. | Resection-injury | *Complement receptor C5aR1 plays an evolutionarily conserved role in successful cardiac regeneration (2018)*^102^ |
| Newt (Notophthalmus viridescens) | Hedgehog, Mycn | Hedgehog (HH)-Gli1-Mycn signaling was essential for CM proliferation in newts, via proliferation of pre-existing CMs. Loss of function in mice and human hiPSC derived CMs reduced proliferative ability. | Resection-injury | *A conserved HH-Gli1-Mycn network regulates heart regeneration from newt to human (2018)*^103^ |
| Axolotl (*Ambystoma mexicanum*) | Immune response | Pro-inflammatory stimuli by injection with LPS or anti-inflammatory stimuli with prednisolone in the pericardial space after injury did not negatively affect heart regeneration | Cryoinjury | *Cardiac regeneration in the axolotl is unaffected by alterations in leukocyte numbers induced by lipopolysaccharide and prednisolone (2021)*^43^ |
| Newt *(Pleurodeles waltl)* | Regeneration | After injury the heart had an increased number of proliferating cells and regenerated anatomically. After injury genes with GO terms: response to wound healing, cell division, muscle system process, and several immune system related terms were upregulated | Cryoinjury | *° procedure-induced cardiac regeneration shows unique gene expression profiles in the newt Pleurodeles waltl (2022)*^104^ |
| Newt *(Pleurodeles waltl)* | Epicardium | Epicardium derived cells migrated to the site of injury and underwent transdifferentiation into CMs in response to injury | Cryoinjury | *Epicardium-derived cells organize through tight junctions to replenish cardiac muscle in salamanders (2022)*^9^ |

**Supplementary Table 2.** **Metabolomics results.** HMDB is accession number for the Human Metabolome Database. Listed P values are from unpaired t-tests comparing each time point to baseline.

| **Metabolomics Results** | | | | | | | |
| --- | --- | --- | --- | --- | --- | --- | --- |
|  | **HMDB ID** |  | **Baseline** | **4 dpi** | **14 dpi** | **30 dpi** | **60 dpi** |
| **Purine Metabolism** | | | | | | | |
| Adenine | HMDB0000034 | **Group Mean** | 1038258 | 568912 | 683384 | 822745 | 1053897 |
|  |  | **Standard deviation** | 89995 | 234257 | 91652 | 144195 | 264187 |
|  |  | **P value** |  | 0.0333 | 0.0030 | 0.0792 | 0.9277 |
| D-Ribose 5-Phosphate | HMDB0001548 | **Group Mean** | 69925 | 34279 | 45234 | 47634 | 60011 |
| R5P |  | **Standard deviation** | 12878 | 8570 | 6779 | 7903 | 11347 |
|  |  | **P value** |  | 0.0095 | 0.0363 | 0.0511 | 0.3563 |
| Deoxyadenosine Monophosphate | HMDB0000905 | **Group Mean** | 3018 | 16117 | 4751 | 3297 | 3555 |
| dAMP |  | **Standard deviation** | 450 | 14881 | 889 | 531 | 592 |
|  |  | **P value** |  | 0.2247 | 0.0345 | 0.5139 | 0.2609 |
| Inosine Monophosphate | HMDB0000175 | **Group Mean** | 183617 | 31883 | 70864 | 99842 | 139907 |
| IMP |  | **Standard deviation** | 48877 | 11864 | 38490 | 55840 | 96650 |
|  |  | **P value** |  | 0.0103 | 0.0216 | 0.0992 | 0.5194 |
| Uric Acid | HMDB0000289 | **Group Mean** | 2592 | 5010 | 1989 | 1936 | 2148 |
|  |  | **Standard deviation** | 304 | 1157 | 302 | 222 | 226 |
|  |  | **P value** |  | 0.0321 | 0.0507 | 0.0262 | 0.0927 |
| Xanthosine Monophosphate | HMDB0001554 | **Group Mean** | 19268 | 5711 | 8617 | 17699 | 20614 |
| XMP |  | **Standard deviation** | 1770 | 1250 | 2364 | 7980 | 8236 |
|  |  | **P value** |  | 0.0001 | 0.0010 | 0.7597 | 0.7984 |
| Glutamine | HMDB0000641 | **Group Mean** | 140335 | 103486 | 78760 | 91032 | 125359 |
|  |  | **Standard deviation** | 30825 | 39137 | 16368 | 20646 | 18856 |
|  |  | **P value** |  | 0.2499 | 0.0318 | 0.0672 | 0.5052 |
| **Pyrimidine Metabolism** | | | | | | | |
| Cytidine Monophosphate | HMDB0000095 | **Group Mean** | 15326 | 12303 | 12853 | 11868 | 13334 |
| CMP |  | **Standard deviation** | 989 | 781 | 2027 | 1261 | 1523 |
|  |  | **P value** |  | 0.0067 | 0.1245 | 0.0107 | 0.1143 |
| Cytidine | HMDB0000089 | **Group Mean** | 51474 | 79640 | 58358 | 39125 | 56167 |
|  |  | **Standard deviation** | 9262 | 14833 | 15664 | 5166 | 4489 |
|  |  | **P value** |  | 0.0382 | 0.5420 | 0.1034 | 0.4706 |
| Cytosine | HMDB0000630 | **Group Mean** | 314549 | 424917 | 359488 | 233680 | 321107 |
|  |  | **Standard deviation** | 18250 | 68768 | 96507 | 30588 | 26764 |
|  |  | **P value** |  | 0.0648 | 0.4824 | 0.0115 | 0.7394 |
| Glutamine | HMDB0000641 | **Group Mean** | 140335 | 103486 | 78760 | 91032 | 125359 |
|  |  | **Standard deviation** | 30825 | 39137 | 16368 | 20646 | 18856 |
|  |  | **P value** |  | 0.2499 | 0.0318 | 0.0672 | 0.5052 |
| Uridine | HMDB0000296 | **Group Mean** | 262398 | 409878 | 336741 | 305086 | 307137 |
|  |  | **Standard deviation** | 22741 | 78688 | 100303 | 85120 | 84134 |
|  |  | **P value** |  | 0.0427 | 0.2918 | 0.4559 | 0.4318 |
| **Pentose Phosphate Pathway** | | | | | | | |
| Arginine | HMDB0000517 | **Group Mean** | 46516 | 36754 | 30150 | 38816 | 53068 |
|  |  | **Standard deviation** | 13317 | 13112 | 7873 | 7651 | 18861 |
|  |  | **P value** |  | 0.4005 | 0.1280 | 0.4266 | 0.6424 |
| D-Ribose 5-Phosphate | HMDB0001548 | **Group Mean** | 69925 | 34279 | 45234 | 47634 | 60011 |
| R5P |  | **Standard deviation** | 12878 | 8570 | 6779 | 7903 | 11347 |
|  |  | **P value** |  | 0.0095 | 0.0363 | 0.0511 | 0.3563 |
| Gluconate | HMDB0000625 | **Group Mean** | 9963 | 12331 | 8056 | 9616 | 8483 |
|  |  | **Standard deviation** | 3156 | 1511 | 1685 | 1875 | 1332 |
|  |  | **P value** |  | 0.3018 | 0.4018 | 0.8765 | 0.4955 |
| Glycerate | HMDB0000139 | **Group Mean** | 10606 | 26999 | 10579 | 16917 | 11775 |
|  |  | **Standard deviation** | 2273 | 3951 | 2975 | 7090 | 5926 |
|  |  | **P value** |  | 0.0018 | 0.9905 | 0.2233 | 0.7663 |
| **Glutathione Metabolism** | | | | | | | |
| Cytidine 2'-3'-Cyclic Phosphate | HMDB0011691 | **Group Mean** | 4599 | 3098 | 3827 | 3424 | 3922 |
|  |  | **Standard deviation** | 490 | 383 | 1370 | 576 | 867 |
|  |  | **P value** |  | 0.0066 | 0.4130 | 0.0369 | 0.2949 |
| Glutathione (Oxidized) | HMDB0003337 | **Group Mean** | 643748 | 1275288 | 780422 | 520062 | 726312 |
|  |  | **Standard deviation** | 72583 | 379121 | 282547 | 203245 | 112737 |
|  |  | **P value** |  | 0.0608 | 0.4701 | 0.3805 | 0.3338 |
| Glutathione (Reduced) | HMDB0000125 | **Group Mean** | 585899 | 291102 | 316181 | 512620 | 675248 |
|  |  | **Standard deviation** | 86437 | 159589 | 24583 | 117305 | 45823 |
|  |  | **P value** |  | 0.0409 | 0.0095 | 0.4200 | 0.1801 |
| Ophthalmic Acid | HMDB0005765 | **Group Mean** | 18883 | 61382 | 23701 | 18081 | 26082 |
|  |  | **Standard deviation** | 4972 | 7752 | 2592 | 6370 | 7884 |
|  |  | **P value** |  | 0.0004 | 0.2029 | 0.8694 | 0.2379 |
| Spermidine | HMDB0001257 | **Group Mean** | 5954 | 10968 | 10451 | 9744 | 15060 |
|  |  | **Standard deviation** | 1025 | 2890 | 2236 | 2535 | 6343 |
|  |  | **P value** |  | 0.0510 | 0.0316 | 0.0750 | 0.0871 |
| **Protein Biosynthesis** | | | | | | | |
| Proline | HMDB0000162 | **Group Mean** | 329237 | 617755 | 341697 | 303367 | 610016 |
|  |  | **Standard deviation** | 44670 | 58372 | 86302 | 21948 | 93748 |
|  |  | **P value** |  | 0.0007 | 0.8340 | 0.4148 | 0.0079 |
| **Glycine, Serine and Threonine Metabolism** | | | | | | | |
| Betaine | HMDB0000043 | **Group Mean** | 408729 | 523967 | 310435 | 342274 | 296319 |
|  |  | **Standard deviation** | 73859 | 127442 | 69312 | 44360 | 35965 |
|  |  | **P value** |  | 0.2355 | 0.1440 | 0.2401 | 0.0717 |
| Creatine | HMDB0000064 | **Group Mean** | 7104186 | 6743039 | 7047303 | 7443327 | 7447579 |
|  |  | **Standard deviation** | 151771 | 182775 | 65603 | 225074 | 150847 |
|  |  | **P value** |  | 0.0401 | 0.5827 | 0.0801 | 0.0320 |
| Glycerate | HMDB0000139 | **Group Mean** | 10606 | 26999 | 10579 | 16917 | 11775 |
|  |  | **Standard deviation** | 2273 | 3951 | 2975 | 7090 | 5926 |
|  |  | **P value** |  | 0.0018 | 0.9905 | 0.2233 | 0.7663 |
| Phosphoserine | HMDB0000272 | **Group Mean** | 114110 | 70687 | 82647 | 69616 | 60901 |
|  |  | **Standard deviation** | 21024 | 4896 | 12726 | 7355 | 8180 |
|  |  | **P value** |  | 0.0340 | 0.0781 | 0.0289 | 0.0159 |
| **Glycerophospholipid and Phingolipid Metabolism** | | | | | | | |
| CDP-Ethanolamine | HMDB0001564 | **Group Mean** | 10047 | 7282 | 7330 | 7552 | 8478 |
|  |  | **Standard deviation** | 925 | 1002 | 2415 | 495 | 710 |
|  |  | **P value** |  | 0.0128 | 0.1454 | 0.0110 | 0.0615 |
| Dipalmitoyl-Phosphatidylcholine | HMDB0000564 | **Group Mean** | 51133 | 13792 | 27382 | 41238 | 33902 |
|  |  | **Standard deviation** | 17091 | 11954 | 8694 | 24797 | 14470 |
|  |  | **P value** |  | 0.0244 | 0.0915 | 0.5925 | 0.2322 |
| Glycerophosphocholine | HMDB0000086 | **Group Mean** | 810508 | 375822 | 693292 | 880896 | 885462 |
|  |  | **Standard deviation** | 179010 | 67282 | 64690 | 76071 | 17552 |
|  |  | **P value** |  | 0.0185 | 0.3496 | 0.5644 | 0.5217 |
| LysoPE(22:6) | HMDB0011496 | **Group Mean** | 839261 | 97473 | 295132 | 411739 | 415226 |
|  |  | **Standard deviation** | 368357 | 130356 | 77495 | 312280 | 219497 |
|  |  | **P value** |  | 0.0334 | 0.0806 | 0.1774 | 0.1487 |
| lysoPE(20:3) | HMDB0011484 | **Group Mean** | 86706 | 19427 | 41196 | 56964 | 48070 |
|  |  | **Standard deviation** | 32094 | 17969 | 10221 | 36105 | 26332 |
|  |  | **P value** |  | 0.0270 | 0.0866 | 0.3278 | 0.1600 |
| LysoPE(20:4) | HMDB0011487 | **Group Mean** | 168006 | 26069 | 61232 | 99517 | 97169 |
|  |  | **Standard deviation** | 67526 | 28281 | 15215 | 68147 | 54602 |
|  |  | **P value** |  | 0.0281 | 0.0682 | 0.2625 | 0.2095 |
| LysoPE(18:2) | HMDB0011477 | **Group Mean** | 44274 | 12511 | 19819 | 30175 | 23762 |
|  |  | **Standard deviation** | 16148 | 6760 | 5407 | 15457 | 7915 |
|  |  | **P value** |  | 0.0345 | 0.0734 | 0.3166 | 0.1135 |
| LysoPE(18:1) | HMDB0011475 | **Group Mean** | 96622 | 20926 | 38077 | 68328 | 52841 |
|  |  | **Standard deviation** | 41104 | 18856 | 7835 | 42912 | 24966 |
|  |  | **P value** |  | 0.0415 | 0.0881 | 0.4411 | 0.1763 |
| Phosphoethanolamine | HMDB0000224 | **Group Mean** | 61911 | 36373 | 43829 | 41191 | 35057 |
|  |  | **Standard deviation** | 9042 | 1861 | 2923 | 5741 | 2821 |
|  |  | **P value** |  | 0.0143 | 0.0348 | 0.0198 | 0.0106 |
| **Histidine Metabolism** | | | | | | | |
| Carnosine | HMDB0000033 | **Group Mean** | 10272 | 2326 | 4213 | 2706 | 3518 |
|  |  | **Standard deviation** | 2649 | 1094 | 1155 | 1006 | 577 |
|  |  | **P value** |  | 0.0087 | 0.0212 | 0.0108 | 0.0190 |
| Histamine | HMDB0000870 | **Group Mean** | 251248 | 194424 | 212795 | 236379 | 252458 |
|  |  | **Standard deviation** | 27573 | 22869 | 6993 | 13389 | 11560 |
|  |  | **P value** |  | 0.0346 | 0.0912 | 0.4446 | 0.9475 |
| Urocanate | HMDB0000301 | **Group Mean** | 198853 | 164316 | 147301 | 178263 | 209404 |
|  |  | **Standard deviation** | 44533 | 34061 | 27868 | 22349 | 18133 |
|  |  | **P value** |  | 0.3297 | 0.1495 | 0.5101 | 0.7233 |
| **Cysteine and Methionine Metabolism** | | | | | | | |
| 5'-Methylthioadenosine | HMDB0001173 | **Group Mean** | 78739 | 141073 | 99084 | 47637 | 77714 |
|  |  | **Standard deviation** | 11249 | 26433 | 25682 | 13565 | 21602 |
|  |  | **P value** |  | 0.0193 | 0.2755 | 0.0233 | 0.9450 |
| Glyoxylate | HMDB0000119 | **Group Mean** | 13924 | 11681 | 10778 | 11037 | 13720 |
|  |  | **Standard deviation** | 1020 | 570 | 1655 | 861 | 1566 |
|  |  | **P value** |  | 0.0229 | 0.0379 | 0.0101 | 0.8571 |
| Phosphoserine | HMDB0000272 | **Group Mean** | 114110 | 70687 | 82647 | 69616 | 60901 |
|  |  | **Standard deviation** | 21024 | 4896 | 12726 | 7355 | 8180 |
|  |  | **P value** |  | 0.0340 | 0.0781 | 0.0289 | 0.0159 |
| **Other** | | | | | | | |
| N-Acetylglutamic acid | HMDB0001138 | **Group Mean** | 41590 | 20851 | 35301 | 18648 | 29107 |
|  |  | **Standard deviation** | 13097 | 3060 | 10421 | 5806 | 10159 |
|  |  | **P value** |  | 0.0678 | 0.5404 | 0.0483 | 0.2427 |
| Malate | HMDB0000156 | **Group Mean** | 33490 | 44104 | 43706 | 29105 | 41503 |
|  |  | **Standard deviation** | 3054 | 4937 | 9661 | 7060 | 10227 |
|  |  | **P value** |  | 0.0249 | 0.1637 | 0.3782 | 0.2719 |
| Uridine Diphosphate Glucose | HMDB0000286 | **Group Mean** | 4428 | 7109 | 5774 | 6682 | 4811 |
|  |  | **Standard deviation** | 1173 | 1187 | 1484 | 509 | 651 |
|  |  | **P value** |  | 0.0319 | 0.2661 | 0.0368 | 0.6429 |
| N-N-N-Trimethyllysine | HMDB0001325 | **Group Mean** | 28510 | 39438 | 28705 | 31661 | 29620 |
|  |  | **Standard deviation** | 5834 | 4767 | 3249 | 4559 | 1929 |
|  |  | **P value** |  | 0.0473 | 0.9618 | 0.4904 | 0.7715 |

**Supplementary Table 3. Source of material and data points in main experiment group.**

| Group | Subgroups (named according to time of tissue harvest) | Echocardiography (heart function). Fig 1a-c. | Echocardiography (non-contracting fraction). Fig 1d.  *NB! Included animals measured repeatedly at all included time-points* | Histology (Infarction fraction). Fig 1e. | Respirometry. Fig 2a.  *NB! Included animals measured repeatedly at all included time-points* | Lactate. Fig 2b.  *NB! Included animals measured repeatedly at all included time-points* | Auto-radiography.  Fig 4. | IF. Fig 8. | Enzyme-stain. Fig 9. |
| --- | --- | --- | --- | --- | --- | --- | --- | --- | --- |
| Main experiment (n = 24) | Baseline/ uninjured (n = 4)  D4 (n = 4)  D14 (n = 4)  D30 (n = 4)  D60 (n = 4)  D120 (n = 4) | -  -  -  -  -  × | *****  ******  ×  ×  ******  × | *****  ×  ×  ×  ×  × | -  -  -  -  -  × | -  -  -  -  -  × | ×  ×  ×  ×  ×  × | ×  ×  ×  ×  ×  - | ×  ×  ×  ×  ×  - |
| -= not included, × = included, * not included because uninjured animals have no injury size to measure, ** not included due to a technical problem with the ultrasound machine at the correct time of imaging. | | | | | | | | | |

**Supplementary Table 4. Chemicals and Reagents.**

**REFERENCES FOR SUPPLEMENTARY MATERIALS**

S1. Smith, C.A., Want, E.J., O'Maille, G., Abagyan, R., and Siuzdak, G.. XCMS: Processing mass spectrometry data for metabolite profiling using nonlinear peak alignment, matching, and identification. *Analytical Chemistry. 2006; 78*:779-787.

S2. Tautenhahn, R., Böttcher, C., and Neumann, S. Highly sensitive feature detection for high resolution LC/MS. BMC *Bioinformatics.* 2008; *9*:504.

S3. Prince, J.T., and Marcotte, E.M. Chromatographic Alignment of ESI-LC-MS Proteomics Data Sets by Ordered Bijective Interpolated Warping. *In Analytical Chemistry (American Chemical Society)*. 2006; 6140-6152.

S4. Kuhl, C., Tautenhahn, R., Böttcher, C., Larson, T.R., and Neumann, S. CAMERA: An Integrated Strategy for Compound Spectra Extraction and Annotation of Liquid Chromatography/Mass Spectrometry Data Sets. *In Analytical Chemistry (American Chemical Society).* 2012; 283-289.

S5. Benjamini, Y. and Hochberg, Y. Controlling the False Discovery Rate: A Practical and Powerful Approach to Multiple Testing. *Journal of the Royal Statistical Society: Series B (Methodological).* 1995; 57:289-300.

S6. Shen, X., Wu, S., Liang, L., Chen, S., Contrepois, K., Zhu, Z.-J., and Snyder, M. metID: an R package for automatable compound annotation for LC−MS-based data. *Bioinformatics.* 2021; *38*:568-569.
